# A Clinical and Physiological Prospective Observational Study on the Management of Pediatric Shock in the Post-Fluid Expansion as Supportive Therapy Trial Era

**DOI:** 10.1097/PCC.0000000000002968

**Published:** 2022-04-21

**Authors:** Nchafatso G. Obonyo, Peter Olupot-Olupot, Ayub Mpoya, Julius Nteziyaremye, Martin Chebet, Sophie Uyoga, Rita Muhindo, Jonathon P. Fanning, Kenji Shiino, Jonathan Chan, John F. Fraser, Kathryn Maitland

**Affiliations:** 1 Kenya Medical Research Institute, Clinical Sciences Department, Wellcome Trust Research Programme, Kilifi, Kenya; 2 Initiative to Develop African Research Leaders, Kilifi, Kenya; 3 Mbale Clinical Research Institute, Department of Paediatrics, Mbale, Uganda; 4 Critical Care Research Group, The Prince Charles Hospital, Brisbane, VIC, Australia; 5 Faculty of Medicine, University of Queensland, Brisbane, VIC, Australia; 6 School of Medicine, Griffith University, Gold Coast, QLD, Australia; 7 Busitema University, Faculty of Health sciences, Mbale, Uganda; 8 Department of Infectious Disease and Institute of Global Health and Innovation, Division of Medicine, Imperial College, London, United Kingdom

**Keywords:** cardiac biomarkers, echocardiography, fluid resuscitation, management, pediatrics, shock

## Abstract

**Objectives:**

Fluid bolus resuscitation in African children is harmful. Little research has evaluated physiologic effects of maintenance-only fluid strategy.

**Design:**

We describe the efficacy of fluid-conservative resuscitation of septic shock using case-fatality, hemodynamic, and myocardial function endpoints.

**Setting:**

Pediatric wards of Mbale Regional Referral Hospital, Uganda, and Kilifi County Hospital, Kenya, conducted between October 2013 and July 2015. Data were analysed from August 2016 to July 2019.

**Patients:**

Children (≥ 60 d to ≤ 12 yr) with severe febrile illness and clinical signs of impaired perfusion.

**Interventions:**

IV maintenance fluid (4 mL/kg/hr) unless children had World Health Organization (WHO) defined shock (≥ 3 signs) where they received two fluid boluses (20 mL/kg) and transfusion if shock persisted. Clinical, electrocardiographic, echocardiographic, and laboratory data were collected at presentation, during resuscitation and on day 28. Outcome measures were 48-hour mortality, normalization of hemodynamics, and cardiac biomarkers.

**Measurement and main Results:**

Thirty children (70% males) were recruited, six had WHO shock, all of whom died (6/6) versus three of 24 deaths in the non-WHO shock. Median fluid volume received by survivors and nonsurvivors were similar (13 [interquartile range (IQR), 9–32] vs 30 mL/kg [28–61 mL/kg], *z* = 1.62, *p* = 0.23). By 24 hours, we observed increases in median (IQR) stroke volume index (39 mL/m^2^ [32–42 mL/m^2^] to 47 mL/m^2^ [41–49 mL/m^2^]) and a measure of systolic function: fractional shortening from 30 (27–33) to 34 (31–38) from baseline including children managed with no-bolus. Children with WHO shock had a higher mean level of cardiac troponin (*t* = 3.58; 95% CI, 1.24–1.43; *p* = 0.02) and alpha-atrial natriuretic peptide (*t* = 16.5; 95% CI, 2.80–67.5; *p* < 0.01) at admission compared with non-WHO shock. Elevated troponin (> 0.1 μg/mL) and hyperlactatemia (> 4 mmol/L) were putative makers predicting outcome.

**Conclusions:**

Maintenance-only fluid therapy normalized clinical and myocardial perturbations in shock without compromising cardiac or hemodynamic function whereas fluid-bolus management of WHO shock resulted in high fatality. Troponin and lactate biomarkers of cardiac dysfunction could be promising outcome predictors in pediatric septic shock in resource-limited settings.

International pediatric septic shock treatment guidelines have recently been updated recommending fluid boluses of between 40 and 60 mL/kg given over 1 hour in settings with access to intensive care treatments. Whereas in settings without access to intensive care fluid boluses are restricted to children with hypotension, providing 10-20 mL/kg of isotonic fluids over 1 hour (1). The latter recommendation was based on the evidence provided from the multicentre Phase III randomized controlled trial of fluid resuscitation in severe febrile illness (Fluid Expansion As Supportive Therapy [FEAST]) which demonstrated a 45% relative increased risk of mortality with fluid bolus (with albumin or saline) compared with nonbolus controls (95% CI, 1.13–1.86; *p* = 0.003) (2, 3). Subsequent analyses indicated the major excess in mortality in the bolus arms were due to cardiogenic shock as the terminal clinical event rather than fluid overload (4). Nevertheless, the 2013 updated World Health Organization (WHO) pediatric inpatient guidelines for resource-limited settings continue to recommend use of fluid boluses for children with all three signs of impaired perfusion (WHO shock criteria): a prolonged capillary refilling time (CRT) greater than 3 seconds, cool peripheries, and a weak and fast pulse (5). These criteria were recommended as a proxy for hypotension since blood pressure measurement is not widely available. We have previously demonstrated that the WHO shock criteria are very rare (~ 0.1%) among general pediatric admission cohorts in resource-limited settings. Mortality in this group, however, was very high (80–100%) (6). These findings question whether the 2013 WHO guidelines for fluid resuscitation are relevant to African children (7).

To date, there have been no clinical physiologic studies investigating no-bolus fluid-conservative strategy and the current WHO shock fluid management guideline. We therefore conducted an observational study on nonmalnourished children admitted with severe febrile illness and impaired perfusion (FEAST trial criteria [2, 3]) managed conservatively with maintenance-only fluid and for children with WHO shock fluid boluses as recommended by the 2013 WHO guidelines (the Management of Paediatric Septic Shock, MAPS study). We also aimed explored baseline clinical indices and biomarkers that could predict mortality.

## Materials and Methods

Ethical approval to conduct the study was sought and obtained from the Kenya Medical Research Institute—Scientific and Ethics Review Unit (2541: from 10 to 10-2013 to 09-10-2014) and the Mbale Regional Referral Hospital—Institutional Review Committee (protocol approval number REIRC 005/2013: from 13 to 03-2013 to 18-11-2019). When prior written consent from parents or legal guardians could not be obtained, ethics committees approved verbal assent with delayed written informed consent as soon as practicable (8).

### Study Design

A prospective observational study was conducted in two hospitals (Mbale Regional Referral Hospital, Uganda, and Kilifi County Hospital, Kenya) in accordance with STrengthening the Reporting of OBservational studies in Epidemiology (STROBE) guidelines (9) between October 2013 and July 2015. Data were analysed from August 2016 to July 2019. As in the FEAST trial, eligible patients included children greater than or equal to 60 days old to less than or equal to 12 years old with severe febrile illness (impaired consciousness and/or respiratory distress) and one or more signs of impaired perfusion (CRT > 3 s, temperature gradient, or weak pulse). Children with severe acute malnutrition, gastroenteritis, or noninfectious causes of shock or known heart disease were excluded.

### Study Procedures, Fluid Management, and Endpoints

Consecutive recruitment of the first 30 eligible and consenting children was done with no formal sample size calculation. Consent process followed that of the FEAST trial (8). All participants had structured clinical assessments, blood/urine samplings, 12-lead electrocardiograms (10–12) and echocardiograms (Vivid.i General Electric Medical Systems with simultaneous electro-cardiogram display) recordings preformed at prespecified timepoints (baseline), postfluid resuscitation (at 8 hr), at 24 hours, and at 1-month follow-up (Methods, Supplementary Digital Content, http://links.lww.com/PCC/C59). Echocardiographic readings were reviewed independently by a cardiologist blinded to clinical status. Children with impaired perfusion received maintenance-only IV fluids (4 mL/kg/hr) as per FEAST control arm followed by oral hydration. Children with WHO shock (≥ 3 criteria) received an initial two 20 mL/kg boluses of saline given over 1 hour. Children with severe anemia (hemoglobin < 5 g/dL) or persisting signs of shock at 8 hours received blood transfusion (20 mL/kg whole blood or 10 mL/kg packed cells) over 3–4 hours as recommended by current WHO guidelines (5).

The endpoints included 48-hour mortality, correction (to normal ranges) of echocardiographic hemodynamic, myocardial performance indices, and cardiac biomarkers measuring evidence of myocardial injury (troponin) (13) and stretch (natriuretic peptides) (14) and adverse events related to the resuscitation.

### Statistical Analysis

All analysis was performed using STATA, Version 15 (StataCorp LLC, College Station, TX). Mean or mean difference (± SD) and median (interquartile range [IQR]) were presented for normally and nonnormally distributed data respectively. Overall normality of data distribution was assessed using the skewness and kurtosis test (sktest). Test for trend over time was performed on variables recorded at admission and repeated after fluid administration until 1-month follow-up. Baseline variables in fatalities versus survivors to assess predictors of outcome and to compare number of existing criteria for shock greater than or equal to three (WHO criteria) or less than or equal to two criteria using a two-sample *t* test (normally distributed data) and Wilcoxon rank-sum test (continuous nonparametric data). Statistical significance was set at *p* value of less than 0.05 for all analyses. Some variables in the univariate analysis had multicollinearity, and Bonferroni's method was used to adjust for multiple comparisons in the multivariate analysis. However, in the final multivariable model, only variables that had shown significance in the univariate analysis were included, none of which were collinear.

## Results

### Recruitment and Baseline Data

Of the 54 children screened, 30 were eligible and followed up for 1 month (Fig. S1, Supplementary Digital Content, http://links.lww.com/PCC/C59). Baseline demographic, anthropometric, clinical, and laboratory data of all patients are summarized in Table 1 comparing survivors versus nonsurvivors. Median age was 23 months (IQR, 17–35 mo), and 70% of the patients (21/30) were males. Malaria was present in 27% (8/30), 10% (3/30) had HIV infection, 40% (12/30) had severe anemia (hemoglobin < 5 g/dL), and 20% (6/30) had WHO shock. Based on the 2005 age-specific Pediatric Sepsis Consensus Conference Criteria (15), tachycardia was present in 77% (23/30), low systolic blood pressure in 50% (15/30), and leukocytosis in 47% (14/30). Table 2 compares baseline variables of all patients with WHO shock versus non-WHO shock. At admission, children with WHO shock criteria were sicker than non-WHO shock, with a higher proportion being hypoxemic (< 90%), 67% versus 4% (*χ*^2^=3.71; *p* = 0.0002), a higher median potassium (*z* = 2.42; *p* = 0.02), lactate (*z* = 2.05; *p* = 0.04), and troponin (*z* = 3.67; *p* = 0.0002) (Table S1, Supplementary Digital Content, http://links.lww.com/PCC/C59, all fatalities and Table S2, Supplementary Digital Content, http://links.lww.com/PCC/C59, trends from admission to 28 d).

### Volume Administered During Initial 24 Hours

At admission, all patients received initial fluid management with IV maintenance-only fluid (4 mL/kg/hr) except those with WHO shock who received fluid bolus therapy; 11 children with severe anemia (hemoglobin < 5 g/dL) at presentation and seven children with persistent shock received blood transfusion (10 mL/kg packed cells or 20 mL/kg whole blood). Overall, median IV volume given was 14 mL/kg/d (IQR, 10–35 mL/kg/d) since many were able to take oral fluids early in admission. The median volume of fluids administered to nonsurvivors was 30 mL/kg (IQR, 28–61 mL/kg) compared with median volume received by survivors 13 mL/kg (IQR, 9–32 mL/kg) (*p* = 0.23). Mean red cell transfusion volume in 11 children with hemoglobin less than 5 g/dL was 9 mL/kg (IQR, 7–11 mL/kg); seven of 11 patients (64%) required greater than or equal to two transfusions. Over 24 hours, the median volume of blood transfused in nonsurvivors was 16 mL/kg (IQR, 8–21 mL/kg) compared with medium volume received by survivors 8 mL/kg (IQR, 6–12 mL/kg) (*p* = 0.24).

### Outcome

By 48 hours, there were nine of 30 fatalities (30%), all occurring less than 40 hours post admission with the majority of seven of nine (78%) occurring by 24 hours (Fig. S2, Supplementary Digital Content, http://links.lww.com/PCC/C59, shows Kaplan-Meier survival at 48 hr and day 28). Six of the fatalities (67%), five of whom died within 6 hours, had WHO shock translating to a case fatality of 6/6 versus 3/24 in children with less than two shock criteria (*χ*^2^=7.27; *p* = 0.007). The clinical narratives of all fatalities are summarized in Table S3 (Supplementary Digital Content, http://links.lww.com/PCC/C59).

### Echocardiography and Electrocardiography

**Figure 1:**
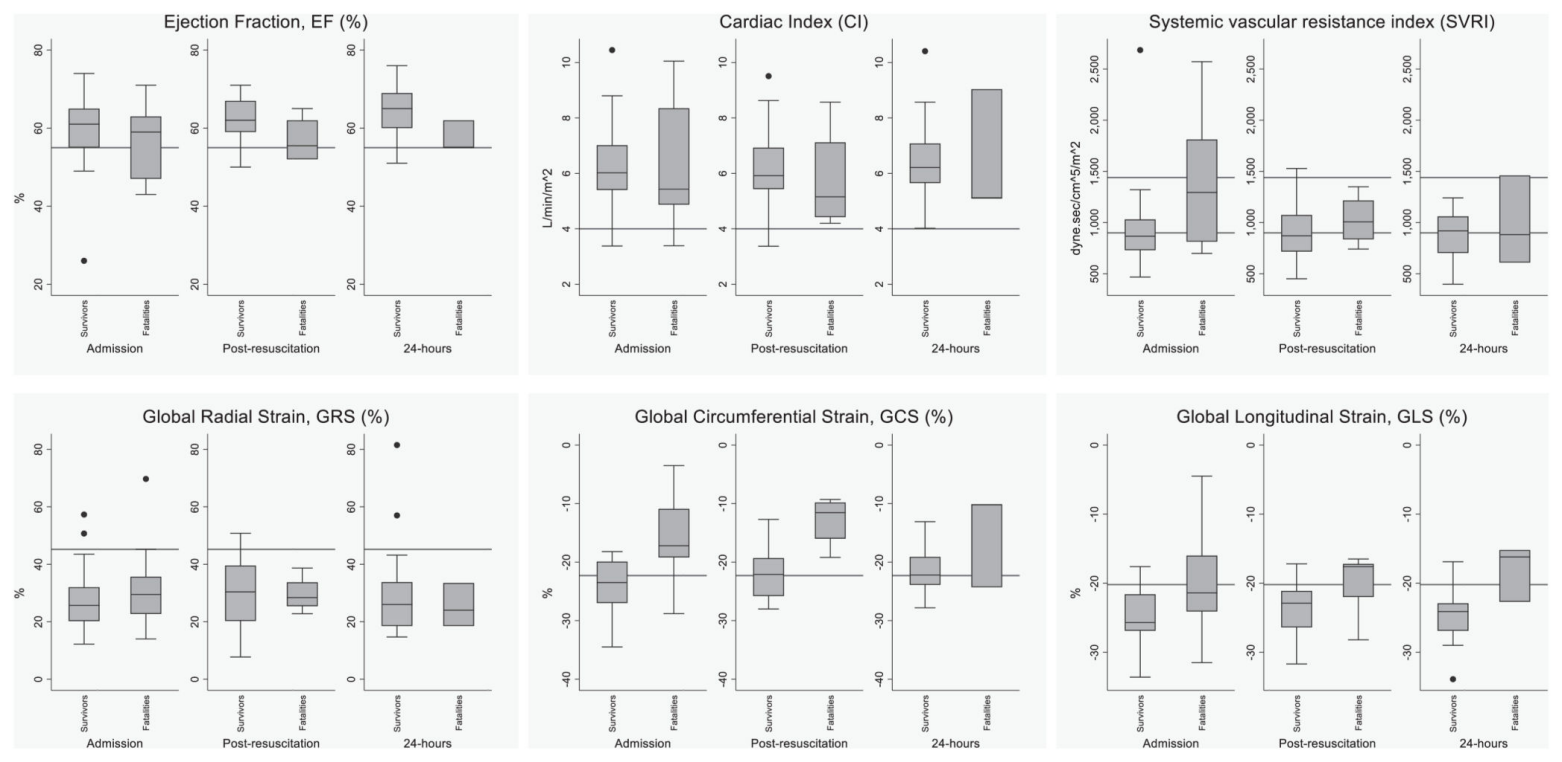
Echocardiographic variables at admission, post resuscitation, and at 24 hr. ***Box*** (displaying the data distribution through their upper and lower quartiles) and ***whiskers plots*** (outliers) of ejection fraction (EF), cardiac index (CI), systemic vascular resistance index (SVRI), global radial strain (GRS), global circumferential strain (GCS), and global longitudinal strain (GLS) comparing survivors versus fatalities. The ***horizontal lines*** indicate published pediatric reference values.

Baseline echocardiographic data are summarized in Table 3, Figure 1, and Figure S3 (Supplementary Digital Content, http://links.lww.com/PCC/C59) shows trends of selected echocardiographic parameters over the first 24 hours. At admission, “Tei index,” a measure of global cardiac function, was reduced to less than or equal to 0.28 in 24 children (80%). The “Tei index” improved to the normal range in 11 survivors (52%) but remained low in 10 survivors (48%). The baseline echocardiographic variables among fatalities comparing WHO shock to non-WHO shock (Table S4, Supplementary Digital Content, http://links.lww.com/PCC/C59). From baseline to 24 hours, we observed increases in volume-sensitive hemodynamic measures increases median (IQR) stroke volume index (SVI) (39 mL/m^2^ [32–42 mL/m^2^] to 47 mL/m^2^ [41–49 mL/m^2^]) and cardiac output (3.0 mL/min [2.4–3.7 mL/min] to 3.1 mL/min [2.8–3.6 mL/min]) and a measure of systolic function fractional shortening median (IQR), 30 (27–33) to 34 (31–38) (Table S5, Supplementary Digital Content, http://links.lww.com/PCC/C59) although these were not statistically different. In the four fatalities, where we had postbaseline echocardiography, fluid volume received was positively associated with increases in SVI (from a median of 36 mL/m^2^ [IQR, 26–47 mL/m^2^] to 41 mL/m^2^ [IQR, 26–48 mL/m^2^]) after receiving a median of 30 mL/kg (IQR 28–61 mL/kg) suggesting fluid responsiveness. Myocardial contractility strain indices measured by speckle tracking in the radial, circumferential, and longitudinal axes were within normal reference limits at admission. Among survivors, median global radial strain (GRS) and global longitudinal strain (GLS) at admission were not significantly higher compared with nonsurvivors (GRS, *z* = 2.88; *p* = 0.05 and GLS, *z* = 3.87; *p* = 0.02). There were no differences in the median GRS and tricuspid annular plane systolic excursion in patients with WHO shock versus those with less than or equal to two shock criteria (*z* = 2.42; *p* = 0.05 and z = 3.14; *p* = 0.02, respectively). Median systemic vascular resistance indices (SVRI) were similar in nonsurvivors and survivors (*z* = 2.29; *p* = 0.09). Baseline electrocardiographic data are summarized in Table S6 (Supplementary Digital Content, http://links.lww.com/PCC/C59) comparing survivors versus non-survivors. There were no significant differences in the baseline electrocardiographic variables at admission between survivors and fatalities or between those with WHO shock versus less than or equal to two shock criteria (Tables S7 and S8, Supplementary Digital Content, http://links.lww.com/PCC/C59).

### Biomarkers Profiles

**Figure 2:**
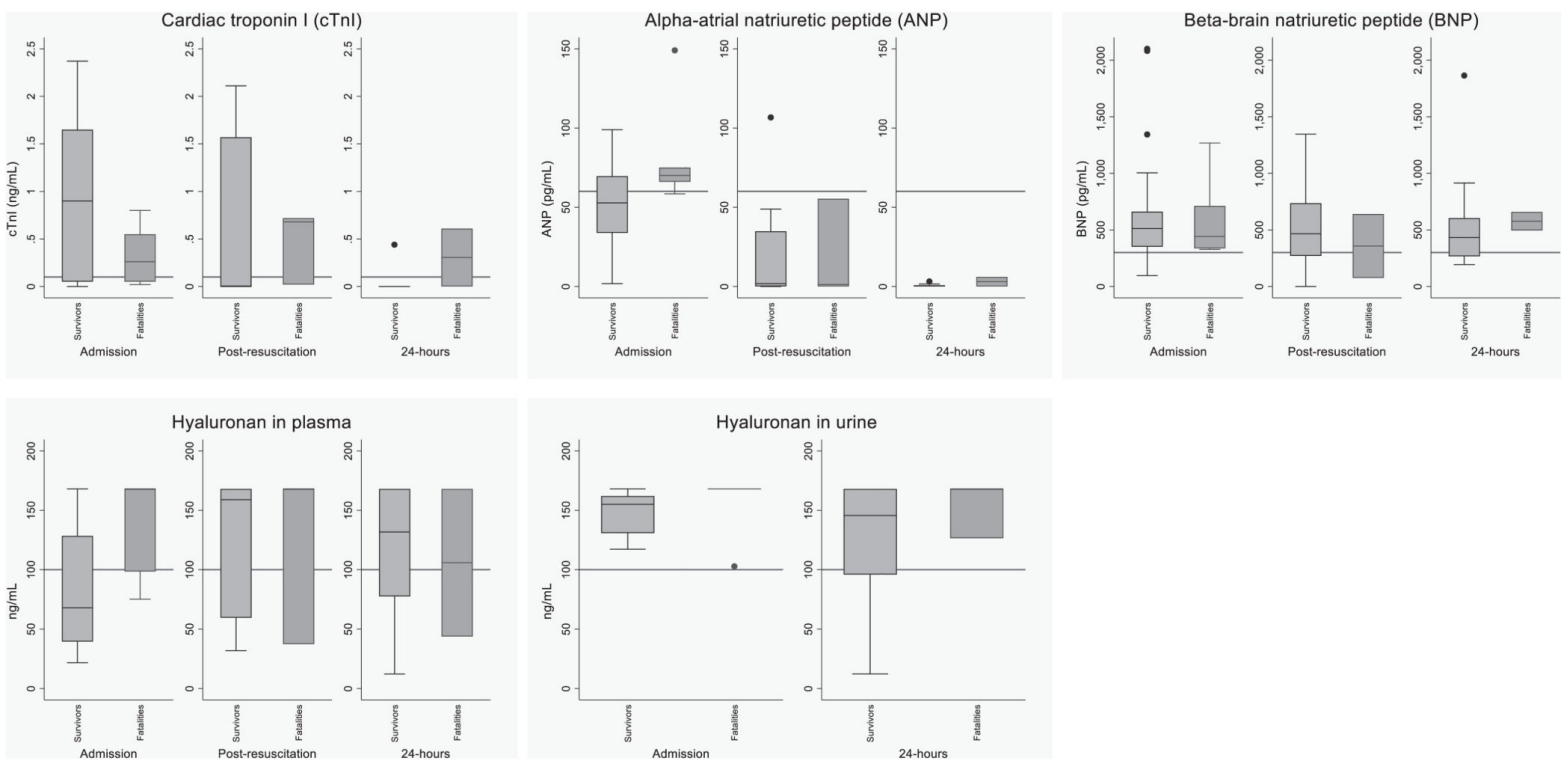
Biomarker profiles at admission, post resuscitation, and at 24 hr. ***Box*** and ***whisker plots*** of cardiac troponin I (cTnI), alpha-atrial natriuretic peptide (ANP), beta-brain natriuretic peptide (BNP), and hyaluronan comparing survivors versus fatalities. The ***horizontal lines*** indicate published reference values.

Figure 2 and Figure S4 (Supplementary Digital Content, http://links.lww.com/PCC/C59) show cardiac biomarker trends over the first 24 hours. At admission, 16 of 30 (53%) had a high cardiac troponin I level (cTnI). In survivors, only two of 23 (9%) had a raised baseline cTnI, but fatal cases had elevated cTnI levels beyond the upper reference limit (≤0.1 μg/mL). Similarly, elevated levels of β-brain natriuretic peptide (BNP; > 300 pg/mL [to convert to nanograms per liter multiply by 1]), α-atrial natriuretic peptide (ANP; > 60 pg/mL [to convert to nanograms per liter multiply by 1]), and plasma hyaluronan (>100 ng/mL [to convert to nmol/L multiply by 2.5]) were present in 70%, 36%, and 40%, respectively, at admission with fatalities having higher median levels of ANP and plasma hyaluronan than survivors (Table S9, Supplementary Digital Content, http://links.lww.com/PCC/C59). At admission, children with WHO shock had higher median cardiac troponin I (cTnI) levels (*t* = 3.58; 95% CI, 1.24–1.43 ng/mL; *p* = 0.02) and alpha-ANP (*t* = 16.5; 95% CI, 2.80–67.5 pg/mL; *p* < 0.01) compared with children less than or equal to two shock criteria. Comparisons of biomarkers among the fatalities with WHO shock versus non-WHO shock are shown in Table S10 (Supplementary Digital Content, http://links.lww.com/PCC/C59) and summaries of biomarker trends over time are summarized in Table S11 (Supplementary Digital Content, http://links.lww.com/PCC/C59).

### Predictors of Mortality

In the univariate analysis, hyperkalemia, hyperlactatemia, hypoglycemia as well as a number of myocardial echocardiographic indices were associated with mortality. The odds ratio (OR) for mortality with an elevated troponin (> 0.1 μg/mL) was 4 (95% CI, 0.63–25.57) (*p* = 0.08) (Table S12, Supplementary Digital Content, http://links.lww.com/PCC/C59). In the multivariable analysis, hyperlactatemia greater than 4 mmol/L was the only variable that showed a significant association with death outcome (OR, 5.38; 95% CI, 4.49-12.71; *p* = 0.003). WHO shock was not included as the composite scoring variable we created introduced instability in the model and was difficult to interpret due to the small sample size.

## Discussion

The MAPS study evaluated feasibility of a fluid conservative strategy in African children with severe febrile illness and shock. Reassuringly, the administration of a maintenance-only fluid management strategy resulted in normalization of the vital indices (heart rate, respiratory rate, CRT, and oxygen saturations) without compromising cardiac function in the cohort of critically ill patients studied. Indeed, it led to a gradual yet significant increase in SVI and other volume-sensitive measures of myocardial function. Majority of the deaths (78% [7/9]) occurred within the first 24 hours. All six children presenting with WHO shock (≥ 3 criteria) and managed as per the current recommended fluid bolus guideline died. Of particular note is that this group had a markedly higher mean level of cardiac troponin I and alpha-ANP compared with those with less than or equal to two shock criteria. Postbaseline measures were not reported in these children as they died before prespecified data/sampling timepoints. In a preclinical trial of ovine endotoxemic shock, fluid bolus resuscitation led to a rise in similar cardiac biomarkers (cTnI and ANP) compared with nonbolus vasopressor support. The rise in ANP was seen in the immediate postfluid resuscitation period whereas that of cTnI was temporally distant peaking 12 hours after the fluid bolus (16).

The WHO shock group of patients represented the sickest patients; in this cohort, five of deaths occurred before the 8-hour timepoint for the clinical and physiologic assessments. The clinical narratives of the fatalities with WHO shock indicated that 3 died during initial bolus therapy, 2 died awaiting an urgent transfusion, and one child had a fatal cardiorespiratory arrest at 16 hours post admission following an initial satisfactory response to fluid boluses. In the FEAST trial, only 65 children had WHO shock (all having prolonged capillary refill time > 3 s, cold extremities, weak and fast pulse). The 48-hour mortality rate in this subgroup was 48% (24/50) who received fluid boluses compared with 20% deaths (3/15) among the nonbolus controls (2, 7). Nevertheless, the WHO guidelines which form the basis for most internationally used guidelines in low-and-middle income countries continue to recommend administration of fluid boluses for this group (17). This was possibly influenced by a systematic review of fluid bolus therapy in resource-limited hospitals by Opiyo et al (18) that considered those with WHO shock as a separate subgroup, concluding that the numbers included in the FEAST trial (*n* = 65) were too small to provide reasonable certainty implying indirectness with respect to evidence. This disregarded the fact that the direction of harm was entirely consistent with the overall analysis; thus, “indirectness” of overall effect should not have been inferred (19). We have previously highlighted that in pediatric hospital admissions in resource-limited hospital, these comprise an exceptionally rare group of children with high fatality (80-100%) (6).

Given the results of the FEAST trial (2, 3) and the recommendations of the systematic review by Ford et al (20), we are concerned that the current recommendations by WHO risk potential harm to a far wider group of children, since it attempts to implement a set of guidelines separating what it considers as “true shock,” that is, the WHO shock definition (three or more signs) from definitions that fewer features of shock which WHO guideline regards as “impaired perfusion” are bound to lead to slippage (17). Unlike in adult guidelines, where hypotension is a prerequisite for defining shock, hypotension in pediatric shock, as demonstrated by the FEAST trial, is a rare and serious event. In the FEAST trial of the 7,838 children with severe febrile illness screened for inclusion, only 29 (0.9%) fulfilled the definition of severe hypotension (FEAST B definition); all received fluid-bolus therapy and had poor outcomes (62% mortality by 48 hr) (3, 6). Notably, the WHO shock definition was not informed by relevant physiologic evidence and based on expert opinion which was intended to identify children with advanced shock including those with hypotension. In a subanalysis of the FEAST trial, we demonstrated that only eight of 29 hypotensive children (27.5%) actually fulfilled the WHO shock definition, and all of those children died (6). In this study, there was no hypotension noted at admission.

A major limitation of the study was the relative small sample size, nevertheless the study represents one of the only pediatric studies to examine myocardial and physiologic linked to fluid management. The major strength of this study is that it was conducted in two centers and involved observation of multiple variables of clinical and hemodynamic function over time. As such, the MAPS study provides physiologic data which reinforce the observations made in the preclinical (RESUS) trial (16) and the FEAST trial (3) that children with WHO shock have extremely deranged myocardial function and biomarkers, and on current recommended management, this universally lead to a fatal outcome, including very elevated levels of cTnI, a myocardial regulatory protein with high specificity for cardiac myocyte damage (independent of volume status) (13). These data therefore corroborate previous findings demonstrating elevated levels of cTnI in septic shock predict left ventricular dysfunction, poor prognosis, and risk of mortality (21–24). Furthermore, we found baseline levels of both ANP and BNP natriuretic peptides were more grossly elevated among patients with WHO shock (≥ 3) criteria compared with those with less than or equal to two shock criteria. Natriuretic peptides classically cause vasodilation, diuresis, and natriuresis and are produced in response to volume or pressure loading on the myocardial chambers, to counteract the excessive cardiac wall stress (25–28). Secretion of ANP also causes increased shedding of the glycocalyx layer lining the vascular endothelia, thus leading to an increase in plasma levels of glycocalyx breakdown products (29). Hyaluronan, a glycosaminoglycans component of the glycocalyx-lining microvascular endothelia, is a sensitive biomarker for vascular damage (30). Our findings of elevated levels of hyaluronan in both plasma and urine, which also increased slightly post-volume resuscitation, supports our original findings that the excess mortality of fluid boluses in the FEAST was mediated through cardiovascular collapse (4).

It could be suggested that echocardiography be used to identify GRS or GLS in order to guide fluid resuscitation; however, this approach remains technically challenging in young pediatric populations on crowded underresourced emergency rooms. Having well-trained pediatricians with requisite skills to conduct and interpret echocardiographic findings also presents a major hurdle. Alternative variables such as hyperlactatemia greater than 4 mmol/L which independently predicted outcome (OR, 5.38; 95% CI, 4.49–12.71; *p* = 0.003) and elevated troponin (> 0.1 μg/mL) could be used in future research studies as potential entry criteria investigating additional strategies to manage cardiovascular compromise.

## Conclusions

This study showed that an elevation in the biomarkers of cardiac and microvascular dysfunction at admission is associated with outcomes, and a fluid-conservative resuscitation strategy using maintenance-only fluid improved clinical signs of shock without compromising cardiac function. We provide physiologic evidence that children presenting with WHO shock (≥ 3) have strong evidence of existing myocardial damage which supports extending conservative fluid management to this group. One major challenge in the future is how to support circulatory collapse and myocardial impairment in resource-limited settings which have no access to high dependency care or ventilatory support. Future research to investigate early inotropic support alongside of judicious use of fluid therapy is warranted.

## Supplementary Material

Supplemental digital content is available for this article. Direct URL citations appear in the printed text and are provided in the HTML and PDF versions of this article on the journal’s website (http://journals.lww.com/pccmjournal).

Supplementary file

## Figures and Tables

**Table 1:** Baseline Characteristics for All Patients, Survivors, and Fatalities

Median (IQR)	All (*N* = 30)	Survivors (*N* = 21)	Fatalities (*N* = 9)	Statistic	*P* ^a^
Sex (% males), *n* (%)	21 (70)	14 (67)	7 (78)	–0.61	0.54
Age (mo)	23 (17–35)	23 (17–37)	26 (18–27)	0.01	0.99
Weight (kg)	10 (10–12)	10 (10–12)	10 (9–11)	0.25	0.81
Height (cm	83 (79–86)	81 (79–87)	85 (82–85)	0.59	0.56
Mid-upper arm circumference (cm)	15 (14–16)	15 (14–16)	14 (13–15)	0.22	0.37
Clinical assessment					
Temperature (°C)	37.9 (37.2–38.4)	37.9 (37.2–38.9)	37.6 (37.0–38.1)	0.16	0.69
Fever (> 39.0°C), *n* (%)	5 (17)	5 (24)	0 (0)	1.61	0.11
Hypothermia (< 36.0°C), *n* (%)	2 (7)	1 (5)	1 (11)	–0.60	0.55
Respiratory rate (breaths/min)	53 (46–60)	54 (46, 60)	50 (49–58)	0.16	0.69
Tachypnea^b^, *n* (%)	30 (100)	21 (100)	9 (100)	NA	NA
O_2_ saturatio*n* (%)	97 (91–98)	97 (94, 98)	95 (83–98)	0.72	0.40
Hypoxemia (<90%), *n* (%)	5 (17)	1 (5)	4 (44)	–2.64	0.008
Heart rate (beats/min)	166 (155–172)	166 (163, 173)	160 (129–169)	0.91	0.34
Tachycardia^c^, *n* (%)	20 (67)	16 (76)	4 (44)	1.69	0.09
Systolic blood pressure (mm Hg)	95 (83–103)	93 (83, 102)	103 (83–109)	1.43	0.23
Hypotension^d^, *n* (%)	0 (0)	0 (0)	0 (0)	NA	NA
Diastolic blood pressure (mm Hg)	53 (51–68)	53 (52, 68)	53 (50–66)	0.06	0.81
Mean arterial pressure (mm Hg)	69 (62–79)	68 (62, 79)	70 (61–78)	1.43	0.23
Capillary refill time > 3 s, *n* (%)	9 (30)	5 (24)	4 (44)	–1.13	0.26
World Health Organization Shock, *n* (%)	6 (20)	0	6 (67)	NA	NA
Laboratory assessment					
Sodium (mmol/L)	133 (132–136)	133 (132-136)	133 (123–136)	0.36	0.55
Potassium (mmol/L)	4.8 (4.2–6.2)	4.7 (4.0–6.1)	5.5 (4.8–7.6)	4.89	0.03
Hyperkalemia (> 5.5 mmol/L), *n* (%)	11 (37)	7 (33)	4 (44)	–0.58	0.56
Creatinine (μmol/L)	53 (35–67)	46 (22, 67)	63 (35–77)	1.76	0.19
Lactate (mmol/L)	3.7 (2.0–11.9)	3.5 (1.9, 11.4)	7.9 (6.4–12.3)	4.89	0.03
Hyperlactatemia (> 4 mmol/L), *n* (%)	12 (40)	6 (29)	6 (67)	–1.95	0.05
Glucose (mmol/L)	8.5 (5.8–9.7)	9.2 (6.6, 10.0)	5.2 (1.4–6.2)	8.00	0.005
Hypoglycemia (< 3 mmol/L), *n* (%)	2 (7)	0 (0)	2 (22)	–2.24	0.03
Hemoglobin (g/dL)	5.4 (4.0–9.6)	5.4 (3.0, 12.0)	5.9 (4.0–9.0)	0.22	0.64
Severe anemia (< 5 g/dL), *n* (%)	12 (40)	9 (43)	3 (33)	0.49	0.63
WBC count × 10^9^/L	15.4 (10.9–23.5)	13.1 (9.0–19.5)	23.2 (15.6–47.9)	4.89	0.03
Leukocytosise *n* (%)	16 (53)	10 (48)	6 (67)	–0.96	0.34
Platelets × 10^9^/L	162 (119–238)	162 (118-264)	142 (119–223)	0.20	0.65
Malaria positive, *n* (%)	8 (27)	5 (24)	3 (33)	–0.54	0.59
HIV positive, *n* (%)	3 (10)	2 (9.5)	1 (11)	–0.13	0.89

IQR = interquartile range, NA = not available.

a *p* comparing medians of baseline characteristics of survivors vs nonsurvivors; univariate analysis with no correction made for multiple comparisons

b Definitions include the following: tachypnea: > 34 breaths/min if < 12 mo old; > 22 breaths/min if > 1 to 5 yr, and > 18 breaths/min if > 5 yr.

c Tachycardia: > 180 beats/min if < 12 mo old; > 160 beats/min if 1–5 yr old, and > 140 beats/min > 5 yr.

d Hypotension: systolic blood pressure of < 50 mm Hg if < 12 mo, < 60 mm Hg if 1–5 yr, and < 70 mm Hg if > 5 yr.

e Leukocytosis: white cell count: > 17.5 × 10^9^/L if < 12 mo, > 15.5 × 10^9^/L if 1–5 yr, and > 13.5 × 10^9^/L if > 5 yr.

Test statistic: χ^2^ test for categorical variables and Mann-Whitney *U* for continuous variables.

**Table 2:** Baseline Characteristic for World Health Organization Shock (≥ 3) and Non-World Health Organization Shock (≤ 2) Criteria

Median (IQR)	WHO Shock (*N* = 6)	Non-WHO Shock (*N* = 24)	Statistic	p^a^
Sex (% males), *n* (%)	5 (83)	16 (67)		
Age (mo)	22 (14–27)	23 (17–38)	–0.60	0.55
Mid-upper arm circumference (cm)	13 (13–15)	15 (14–16)	–2.17	0.03
Temperature (°C)	37.8 (37.4–38.1)	37.9 (37.1–38.9)	–0.57	0.57
Fever (> 39.0°C), *n* (%)	0 (0)	5 (21)	–1.23	0.22
Hypothermia (< 36.0°C), *n* (%)	1 (17)	1 (4)	1.15	0.25
Respiratory rate (breaths/min)	50 (49–64)	54 (44–60)	0.05	0.96
O_2_ saturation (%)	84 (57–97)	98 (95–98)	–2.10	0.04
Hypoxemia (< 90%), *n* (%)	4 (67)	1 (4)	3.71	< 0.001
Heart rate (beats/min)	152 (43–181)	166 (160–172)	–0.32	0.75
Tachycardia,^b^ *n* (%)	3 (50)	17 (71)	–0.98	0.33
Systolic blood pressure (mm Hg)	103 (82–109)	93 (83–103)	0.44	0.66
Hypotension,^c^ *n* (%)	0 (0)	0 (0)	NA	NA
Diastolic blood pressure (mm Hg)	53 (50–66)	57 (52–68)	–0.55	0.58
Mean arterial pressure (mm Hg)	70 (61–80)	68 (62–79)	–0.08	0.94
Capillary refill time > 3 s, *n* (%)	3 (50)	6 (25)	1.20	0.23
Sodium (mmol/L)	130 (123–138)	133 (132–136)	–0.36	0.72
Potassium (mmol/L)	7.0 (5.9–8.3)	4.7 (4.1–6)	2.42	0.02
Hyperkalemia (> 5.5 mmol/L), *n* (%)	4 (67)	7 (29)	1.73	0.08
Creatinine (|jmol/L)	50 (23–71)	53 (38–67)	–0.39	0.70
Lactate (mmol/L)	12.1 (9.9–13.1)	3.6 (1.9–10.8)	2.05	0.04
Hyperlactatemia (> 4 mmol/L), *n* (%)	4 (67)	8 (33)	1.52	0.13
Glucose (mmol/L)	5.3 (1.3–8.2)	8.9 (6.2–9.7)	–1.67	0.09
Hypoglycemia (< 3 mmol/L), *n* (%)	1 (17)	1 (4)	1.15	0.25
Hemoglobin (g/dL)	4.2 (2.5–7.6)	6.5 (4.0–9.6)	–1.02	0.31
Severe anemia (< 5 g/dL), *n* (%)	2 (33)	10 (42)	–0.40	0.69
WBC count × 10^9^/L	23.4 (20.2–37.8)	14.0 (9.0–19.5)	1.78	0.08
Leukocytosis,^d^ *n* (%)	4 (67)	12 (50)	0.75	0.46
Platelets × 10^9^/L	131 (80–254)	167 (126–238)	–0.78	0.43
Malaria positive, *n* (%)	2 (33)	6 (25)	0.40	0.69
HIV positive, *n* (%)	1 (17)	2 (8)	0.66	0.51
Biomarkers				
Troponin (ng/mL)	0.67 (0.61–0.74)	0.09 (0.04–0.26)	3.67	< 0.001
β-brain natriuretic peptide (pg/mL)	798 (563–1,033)	512 (328–1,004)	0.24	0.81
Atrial natriuretic peptide (pg/mL)	111 (91–130)	67 (62–72)	1.08	0.28
Plasma hyaluronan (ng/mL)	168 (168–168)	75 (40–168)	1.78	0.07
Urine hyaluronan (ng/mL)	168 (168–168)	158 (129–168)	2.07	0.04

IQR = interquartile range, NA = not available, WHO = World Health Organization.

a *p* comparing medians of baseline characteristics of patients with WHO shock vs non-WHO shock criteria; univariate analysis with no correction made for multiple comparisons.

b Tachycardia: >180 beats/min if <12 mo of age; >160 beats/min if 1 to 5 yr of age; and >140 beats/min > 5 yr.

c Hypotension: Systolic blood pressure of <50 mm Hg if <12 mo; <60 mm Hg if 1-5 yr; and <70 mm Hg if > 5 yr.

d Leukocytosis: White cell count: >17.5 X 10^9^/L if <12 mo; >15.5 X 10^9^/L if 1-5 yr and >13.5 X 10^9^/L if > 5 yr.

Test statistic: χ^2^ test for categorical variables and Mann-Whitney *U* for continuous variables.

**Table 3:** Baseline Echocardiography Variables at Admission: All Patients, Survivors, and Fatalities

Medians (IQR)	All (*N* = 30)	Survivors (*N* = 21)	Fatalities (*N* = 9)	U^a^	*P*
Tei index	0.15 (0.10–0.23)	0.15 (0.11–0.21)	0.12 (0.10–0.36)	1.67	0.11
Ejection fraction (%)	60 (53–64)	61 (55–66)	59 (47–63)	0.86	0.40
Fractional shortening (%)	30 (27-33)	32 (28-35)	30 (23-32)	1.50	0.19
SV (mL)	19 (16–24)	19 (17–23)	19 (14–24)	0.36	0.97
SV index (mL/m^2^)	39 (31–46)	39 (35–41)	36 (26–47)	0.40	0.76
EDV (mL)	31 (26–42)	31 (27–37)	33 (22–45)	0.39	0.76
EDV index (mL/m^2^)	65 (52–74)	63 (55–69)	68 (41–93)	1.09	0.63
SVR (dynes.s/cm^5^)	433 (394–578)	416 (381–520)	586 (430–791)	2.36	0.08
SVR index (dynes.s/cm^5^/m^2^)	893 (756- 1,271)	865 (720–1,018)	1,231 (811–2,077)	2.29	0.09
Cardiac output (mL/min)	3.0 (2.4–3.7)	3.0 (2.5–3.6)	2.9 (1.8–4.1)	0.25	0.96
Cardiac index (mL/min/m^2^)	6.0 (4.9–7.2)	6.0 (5.4–7.1)	5.4 (4.9–8.4)	0.27	0.96
Inferior vena cava collapsibility index (%)	28 (18–40)	27 (18–39)	35 (21–40)	0.44	0.67
Tricuspid annular plane systolic excursion (mm)	16 (14–18)	16 (15–18)	14 (12–15)	2.53	0.06
Mitral annular plane systolic excursion (mm)	11 (10–12)	11 (10–12)	10 (8–10)	1.97	0.06
Mitral inflow (E′)	0.15 (0.12–0.18)	0.16 (0.13–0.18)	0.11 (0.09–0.15)	2.46	0.06
Early MDF	7.95 (5.86–9.36)	8.11 (6.10–9.14)	7.91 (5.67–9.36)	0.25	0.96
Early to late MDF ratio (E/A)	1.24 (1.11–1.63)	1.22 (1.14–1.35)	1.63 (1.04–1.69)	0.95	0.73
Systolic left ventricular peak velocity	0.08 (0.06–0.09)	0.08 (0.07–0.09)	0.07 (0.05–0.07)	2.34	0.08
Global radial strain (%)	26 (22–32)	41 (29–50)	23 (23–40)	2.88	**0.05**
Global circumferential strain (%)	–21 (–26 to 18)	–19 (–21 to –16)	–21 (–24 to –13)	1.57	0.06
Global longitudinal strain (%)	–24 (–27 to –21)	–22 (–24 to –19)	–13 (–17 to –13)	3.87	**0.02**

E/A = ratio of the flow velocity profile across the mitral valves (E [early filling] velocity wave and late diastole generates a second wave of flow across the valve [A wave], EDV = end diastolic volume, IQR = interquartile range, MDF = diastolic mitral inflow, SV = stroke volume, SVR = systemic vascular resistance.

a Test-statistic: Mann-Whitney *U* test and *p* comparing baseline characteristics of survivors vs fatalities.

## Research in Context

- The 2020 Surviving Sepsis Guidelines recommends that fluid resuscitation in low resource settings should be reserved only for children with hypotension following the publication of the large Phase III trial (Fluid Expansion As Supportive Therapy) which demonstrated excess mortality in children with nonhypotensive shock.
- There has been some reticence to adopt a nobolus strategy, in the absence of physiologic studies demonstrating the mechanism of harm and evidence of correction of cardio-hemodynamic measures in children treated with this fluid-conservative strategy.
- We provide a small, detailed observational study evidence demonstrating the normalization of cardiac and hemodynamic perturbations of shock managed with a no-bolus strategy and evidence of high mortality in children who receiving boluses in accordance with the 2013 World Health Organization pediatric guidelines.

## At the Bedside

- We provide further physiologic and clinical data to support a no-bolus fluid strategy in 34 African children with severe illness and impaired perfusion. Whereas use of the WHO shock guideline resulted in uniform mortality.
- We provide some evidence that bedside markers of cardiac injury and stretch (troponin and ANP) may be of prognostic value.
- Future studies should focus on assessing conservative fluid strategies alongside inotropic support in order to improve outcome in pediatric septic shock.
