# Supplementary material for: A Clinical and Physiological Prospective Observational Study on the Management of Pediatric Shock in the Post-Fluid Expansion as Supportive Therapy Trial Era
Source: Pediatr Crit Care Med. Author manuscript; Available in PMC 2022 Jul 8. (PMC7613033; doi:10.1097/PCC.0000000000002968)
Supplement: Supplementary file [file EMS146167-supplement-Supplementary_file.docx]

## CONTENTS

Table of Contents

[CONTENTS 1](#_Toc98248701)

[SUPPLEMENTARY METHODS 2](#_Toc98248702)

[Study sites 2](#_Toc98248703)

[Inclusion criteria 2](#_Toc98248704)

[Exclusion criteria 2](#_Toc98248705)

[Clinical examination 2](#_Toc98248706)

[Electrocardiogram (ECG) recordings 2](#_Toc98248707)

[Echocardiogram (ECHO) recordings 2](#_Toc98248708)

[Blood and urine sampling 3](#_Toc98248709)

[SUPPLEMENTARY TABLES 5](#_Toc98248710)

[SUPPLEMENTARY FIGURES 17](#_Toc98248711)

[REFERENCES 25](#_Toc98248713)

# SUPPLEMENTARY METHODS

## Study sites

The study was conducted on the pediatric ward at the Mbale Regional Referral Hospital in Mbale, Uganda and the high dependency unit at the Kilifi County Hospital in Kilifi, Kenya.

## Inclusion criteria

- Children ≥60 days to ≤12 years old
- Severe febrile illness presenting to hospital with impaired consciousness (prostration or coma) and/or respiratory distress (deep ‘acidotic’ breathing or in-drawing) and one or more signs of impaired perfusion:
  - Prolonged capillary refilling time (CRP) ≥3 seconds,
  - Cold peripheries (temperature gradient from toe to shin)
  - Weak and rapid radial pulse volume

## Exclusion criteria

- Assent/consent refusal by the parent/guardian
- Severe acute malnutrition:
  - Weight-for-height z-score <-3 of the WHO growth standard or
  - Mid-upper arm circumference (MUAC) measurement of <11.5cm or
  - Kwashiorkor
- Gastroenteritis (hypovolemic shock)
- Non-infectious causes of shock such as trauma, surgery or burns
- known heart disease or found to have an existing congenital cardiac condition on the first echocardiographic examination.

## Clinical examination

Each patient had baseline anthropometry, vital signs and a full clinical assessment recorded on standardized case-record forms. Bedside observations were repeated half-hourly during the fluid resuscitation phase and then hourly thereafter for the next 8-hours and thereafter 4-hourly to 24-hours, followed by daily assessment to discharge and returned for follow-up at day 28 post admission. All other treatment administered including antibiotics, supplementation of oxygen and glucose, blood transfusion for low hemoglobin where indicated were done according to national or WHO guidelines.

## Electrocardiogram (ECG) recordings

Electrocardiographic assessment (Cardiac Acquisition Module, CAM-14, General Electric Medical Systems^®^ and CardioSoft™ v6.51 Diagnostic System Software) was performed at admission (baseline), at 24-hours and at 1-month follow-up. A standard twelve-lead ECG recorded at 25mm/sec was used to collect data on the PR and RR-intervals, QRS-complex duration, QT-interval and QT-dispersion based on validated techniques ^1^. Corrected QT-interval and QT-dispersion adjusted to heart rate (HR) were calculated using Bazett’s formula ^2,3^.

## Echocardiogram (ECHO) recordings

Echocardiographic assessment (Vivid.i General Electric Medical Systems^®^ with simultaneous ECG display) was performed at admission (baseline), post-fluid resuscitation (at 8 hours), at 24-hours and at 1-month follow-up. Data was collected as 3-beat ECG-gated loops and averaged over three cardiac cycles in the subcoastal, apical and parasternal windows using a 6S-RS pediatric cardiac phased-array transducer (GE Healthcare™) based on validated methodology informed the recommendations for quantification methods during the performance of a pediatric echocardiogram: a report from the Pediatric Measurements Writing Group of the American Society of Echocardiography Pediatric and Congenital Heart Disease Council ^4^.

The most commonly used parameters of global left systolic function includes the volumetric ejection fraction (EF), which is effected by loading conditions (volume). The ejection fraction EF was calculated from the equation EF = (end-diastolic volume - end-systolic volume)/end-diastolic volume).

Stroke volume (SV) was derived from the difference between the end-diastolic volume (EDV) and the end-systolic volume (ESV). Fractional shortening (FS) was measured as the percentage change in LV diameter during systole and diastole using the formula ^4^: FS = [(LVEDD – LVESD)/LVEDD] X 100.

Cardiac output (CO) was obtained by multiplication of the stroke volume (SV) with the heart rate (HR) recorded on the ECG.

The measurements of SV, EDV, CO and SVR were all indexed to the patients’ body surface area (BSA) derived in the *Vivid.i* echocardiogram using the configured Haycock formula ^5^: BSA (m^2^) = 0.024265 x weight (kg) ^0.5378^ x height (cm) ^0.3964^

Overall myocardial performance was based on the *Tei* index, a measure of global cardiac function ^6-9^. The modified Simpson’s algorithm using a combination of apical, parasternal short-axis (PSAX) and long-axis (PLAX) views was used to calculate left ventricular (LV) diastolic and systolic volumes ^4,10-12^.

Myocardial deformation was assessed by speckle tracking and quantification of the mean global radial, circumferential and longitudinal strain was performed using automated function imaging (AFI) ^13,14^, in EchoPac™ ver.201 (GE Healthcare™).

The systemic vascular resistance (SVR) was calculated using Darcy’s formula that relates pressure, flow and resistance by dividing the mean blood pressure by the aortic velocity time integral (VTI) flow. Mean arterial pressure was derived from measured systolic and diastolic blood pressure values and height and weight data. Systemic Vascular Resistance Index was automatically calculated using the following parameters: SVR = Mean blood pressure/Velocity Time Integral_aorta ._ Cut-offs for normality of haemodynamic parameters were derived using the data collected by Cattermole and stratified by height rather than age.^15^ The inferior vena cava collapsibility index (IVCCI) served as a surrogate marker of intravascular filling status and was calculated as IVCCI= (IVCmax – IVCmin)/IVCmax)) x 100**.** Z-score regression equations derived by Peterson et al were applied to our data for all intracardiac dimensions.^16^

## Blood and urine sampling

Routine blood sampling was performed at admission (baseline) for clinical investigations, 8-hours, 24-hours, 48-hours and at 1-month follow-up. At the timepoints within the first 24-hours, an additional 0.3mL aliquot of plasma was stored for batch-analysis of cardiac biomarkers (troponin I (cTnI), alpha-atrial natriuretic peptide (ANP), and brain natriuretic peptide (BNP) as well as the microvascular biomarker hyaluronan. Biomarker assays were performed using ELISA kits (ThermoFisher Scientific™, for ANP and BNP; Abcam®, for cTnI; R and D Systems®, for hyaluronan). A 2.0mL sample of urine was collected and stored at admission and at 24-hours for batch-analysis of renal clearance of hyaluronan (R and D Systems® ELISA).

Pediatric biomarker references were based on published literature for troponin (>0.1 µg/L ^17^), β-BNP (>300 pg/ml ^18^) and α-ANP (>60pg/ml ^19,20^). There are no published reference values for plasma or urine hyaluronan in the pediatric population. In adults, the normal mean hyaluronan level in plasma was recently shown to be 24 ± 8 ng/mL ^21^, with levels exceeding 100 ng/mL suggestive of glycocalyx damage ^22^.

# SUPPLEMENTARY TABLES

**Table S1:** Admission clinical and laboratory features of fatalities Table showing comparison of baseline clinical and salient laboratory parameters among patients who died based on the number of shock criteria present at admission

| **n (%) or Medians (IQR)** | **Fatalities (n, 9)** | **≥3 criteria (n, 6)** | **≤2 criteria (n, 3)** | **Test Statistic** | **p*** |
| --- | --- | --- | --- | --- | --- |
| **Biodata and anthropometry** |  |  |  |  |  |
| Sex (% males), n (%) | 7 (78%) | 5 (83%) | 2 (67%) |  |  |
| Age (months) | 26 (18 – 27) | 22 (14 – 27) | 24 (24 – 36) | 0.98 | 0.36 |
| Weight (kg) | 10 (9 – 11) | 10 (8 – 11) | 11 (11 – 13) | 0.97 | 0.38 |
| Height (cm) | 85 (82 – 85) | 82 (78 – 83) | 85 (83 – 93) | 0.78 | 0.49 |
| MUAC (cm) | 14 (13 – 15) | 13 (13 – 15) | 15 (14 – 15) | 0.69 | 0.52 |
| **Clinical assessment** |  |  |  |  |  |
| Temperature (^0^C) | 37.6 (37.0, 38.1) | 37.8 (37.4, 38.1) | 37.0 (36.6, 38.1) | 0.53 | 0.47 |
| Fever (>39.0^0^C), n (%) | 0 (0) | 0 (0) | 0 (0) | N/A | N/A |
| Hypothermia (<36.0^0^C), n (%) | 1 (11) | 1 (17) | 0 (0) | 0.75 | 0.45 |
| Respiratory rate (breaths/min) | 50 (49, 58) | 50 (49, 64) | 58 (38, 58) | 0.19 | 0.66 |
| Tachypnoea**, n (%) | 9 (100) | 6 (100) | 3 (100) | N/A | N/A |
| Oxygen saturation (%) | 95 (83, 98) | 84 (57, 97) | 98 (95, 98) | 2.67 | 0.10 |
| Hypoxemia (<90%), n (%) | 4 (44) | 4 (67) | 0 (0) | 1.90 | 0.06 |
| Heart rate (beats/min) | 160 (129, 169) | 152 (43, 181) | 160 (129, 164) | 0.67 | 0.41 |
| Tachycardia^╬^, n (%) | 4 (44) | 3 (50) | 1 (33) | 0.47 | 0.64 |
| Systolic blood pressure (mmHg) | 103 (83, 109) | 103 (82, 109) | 103 (83, 117) | 0.06 | 0.81 |
| Hypotension^#^, n (%) | 0 (0) | 0 (0) | 0 (0) | N/A | N/A |
| Diastolic blood pressure (mmHg) | 53 (50, 66) | 53 (50, 66) | 65 (43, 68) | 0.19 | 0.66 |
| Mean arterial pressure (mmHg) | 70 (61, 78) | 70 (61, 80) | 71 (56, 78) | 0.53 | 0.47 |
| CRT >3 sec, n (%) | 4 (44) | 3 (50) | 1 (33) | 0.47 | 0.64 |
| **Salient Laboratory parameters** |  |  |  |  |  |
| Potassium (mmol/L), median (IQR) | 5.5 (4.8, 7.6) | 7.0 (5.9, 8.3) | 4.8 (4.4, 5.0) | 2.61 | 0.014 |
| Lactate (mmol/L), median (IQR) | 7.9 (6.4, 12.3) | 12.1 (9.9, 13.1) | 6.4 (3.2, 7.9) | 1.96 | 0.03 |
| Red blood cell count x 10^12^/L, median (IQR) | 1.3 (0.6, 3.8) | 0.7 (0.6, 1.0) | 3.8 (1.6, 5.2) | 3.03 | 0.02 |

Test Statistic: χ^2^**-**test for categorical variables and Mann Whitney, U, for continuous variables

* p-value comparing medians of baseline characteristics of patients with ≥3 *versus* ≤2 shock criteria

Tachypnoea: >34 brpm if <12 months of age; >22 brpm if >1 to 5 yrs and >18 brpm if >5 yrs

^╬^Tachycardia: >180 bpm if <12 months of age; >160 bpm if 1 to 5 years of age; and >140 bpm >5 yrs

^#^Hypotension: Systolic blood pressure of <50 mmHg if <12 months; <60 mmHg if 1-5 years; and <70 mmHg if >5 yrs

^+^Leukocytosis: White cell count: >17.5 X 10^9^/L if <12 months; >15.5 X 10^9^/L if 1-5 years and >13.5 X 10^9^/L if >5 years

**Table S2:** Clinical and laboratory variables over the course of follow-up. Table showing trend of clinical and laboratory parameters at admission, post-fluid resuscitation, at 24-hours and day-28

| **n (%) or Medians (IQR)** | **Pre-fluid**  **(n, 30)** | **Post-fluid**  **(n, 25)** | | **24 hours**  **(n, 23)** | **Day 28**  **(n, 21)** | | **F*** | | **p-trend*** | |
| --- | --- | --- | --- | --- | --- | --- | --- | --- | --- | --- |
| **Clinical assessment** |  |  | |  |  | |  | |  | |
| Temperature (^0^C), | 37.9 (37.2, 38.4) | 37.1 (36.9, 38.1) | | 36.9 (36.1, 37.9) | 36.6 (36.2 - 36.7) | | -4.72 | | <0.0001 | |
| Fever (>39.0^0^C), n (%) | 5 (17) | 1 (4) | | 3 (13) | 0 (0) | |  | |  | |
| Respiratory rate (brpm) | 53 (46 - 60) | 50 (40 - 58) | | 35 (32 - 46) | 34 (32 - 42) | | -4.71 | | <0.0001 | |
| Oxygen saturation (%) | 97 (91 - 98) | 97 (96 - 98) | | 98 (97 - 99) | 99 (99 - 99) | | -4.60 | | <0.0001 | |
| Hypoxemia (<90%), n (%) | 5 (17) | 2 (8) | | 1 (4) | 0 (0) | |  | |  | |
| Heart rate (bpm) | 166 (155 - 172) | 154 (134 - 164) | | 139 (123 - 148) | 115 (109 - 125) | | -5.72 | | <0.0001 | |
| Tachycardia^╬^, n (%) | 20 (67) | 9 (36) | | 2 (9) | 0 (0) | |  | |  | |
| SBP (mmHg) | 95 (83 - 103) | 98 (91 - 103) | | 97 (91 - 103) | 100 (92 - 108) | | 1.09 | | 0.27 | |
| Hypotension^#^, n (%) | 0 (0) | 1 (4) | | 0 (0) | 0 (0) | |  | |  | |
| DBP (mmHg) | 53 (51 - 68) | 62 (53 - 66) | | 62 (56 - 68) | 65 (59 - 70) | | 2.13 | | 0.03 | |
| MAP (mmHg) | 69 (62 - 79) | 76 (72 - 80) | | 74 (65 - 81) | 79 (76 - 81) | | 2.71 | | 0.007 | |
| CRT >3 sec, n (%) | 9 (30) | 1 (4) | | 0 (0) | 0 (0) | |  | |  | |
| **Laboratory assessment** |  | |  |  | |  | |  | |  |
| Sodium (mmol/L) | 133 (132 - 136) | 134 (133 - 136) | | 134 (130 - 137) | 136 (134 - 139) | | 1.63 | | 0.10 | |
| Potassium (mmol/L) | 4.8 (4.2 - 6.2) | 4.9 (4.2 - 6.0) | | 5.1 (3.9 - 6.5) | 4.3 (4.1 - 5.2) | | -0.76 | | 0.45 | |
| Creatinine (µmol/L) | 53 (35 - 67) | 40 (21 - 51) | | 32 (20 - 54) | 31 (13 - 59) | | -1.88 | | 0.06 | |
| Lactate (mmol/L) | 3.7 (2.0 - 11.9) | 2.2 (1.5 - 5.5) | | 1.8 (1.2 - 2.9) | 2.1 (1.2 - 2.7) | | -3.53 | | <0.0001 | |
| Lactate>4mmol/L, n (%) | 12 (40) | 10 (40) | | 4 (17) | 1 (5) | |  | |  | |
| WCC (x10^9^/L) | 15.4 (10.9-23.5) | 13.8 (8.5, 20.9) | | 10.5 (7.5, 21.9) | 7.1 (6.3 - 9.4) | | -2.75 | | 0.006 | |
| Leukocytosis^+^ n (%) | 16 (53) | 11 (44) | | 7 (30) | 1 (5) | |  | |  | |
| Hemoglobin (g/dL) | 5.4 (4.0 - 9.6) | 7.7 (6.5 - 9.5) | | 8.1 (6.5 - 8.7) | 8.9 (6.8 - 11.4) | | 2.27 | | 0.02 | |
| Severe anemia (<5g/dL), n (%) | 12 (40) | 0 (0) | | 1 (4) | 1 (5) | |  | |  | |

See definition in Supplementary Table 1 in Addition SBP (systolic blood pressure), DBP (diastolic blood pressure) and MAP (mean arterial pressure).

*F-statistic and p-value of trend test from baseline to 1-month follow-up

| Tables S3: Clinical Narratives of Fatal cases | | | | | |
| --- | --- | --- | --- | --- | --- |
| WHO-Shock | | |  |  |  |
| Patient ID. 602 (KS) | The child is a 3 years 8 months old male who had been treated at a peripheral facility having presented with history of cough and fever for 3 days but did not improve. He was referred to the hospital for further investigations and medical management.  On presentation, he was febrile (37.9^0^C), tachypnoeic (64 breaths/min), tachycardic (151 beats/min), cyanosed with oxygen saturations of 57% on room air and MAP of 43mmHg. He was dehydrated and had all clinical features of WHO shock present ( prolonged capillary refill time >3 seconds, weak & rapid pulse, cool extremities with temperature gradient from toe-to-shin and impaired level of consciousness: comatose).  Investigations at admission: Severe anaemia (Hb 3mg/dL), lactic acidosis (12.3mmol/L), leucocytosis (WBC count 23.5 X 10^6^/L) and a positive rapid diagnostic test for malaria. His random blood sugar test at admission was 5.3mmol/L.  Fluid management: As per guidelines he received two boluses of normal saline 20mls/kg each and blood transfusion 10mls/kg but died within 2-hours of hospital presentation when still receiving the transfusion and third fluid bolus. | | | | |
| Patient ID. 603 (PM) | The child is a 1 year 6 months old male who had fever and cough for 4 days prior to admission, did not improve with treatment purchased over the counter and developed difficulty in breathing leading to the admission.  On presentation, his axillary temperature was 35.3^0^C he was tachypnoeic (66 breaths/min), tachycardic (181 beats/min), had 97% oxygen saturation in room air and MAP of 60mmHg. He had all clinical features of WHO shock present (prolonged capillary refill time >3 seconds, weak & rapid pulse, cool extremities with temperature gradient from toe-to-shin and impaired level of consciousness: prostration).  Investigations at admission: Severe anaemia (Hb 2.8mg/dL), lactic acidosis (7.9mmol/L), hyponatraemia (123mmol/L), hyperkalaemia (7.6mmol/L), hypoglycaemia 1.3mmol/L) and a positive rapid diagnostic test for malaria.  Fluid management: He received emergency resuscitation and treatment for the hypoglycaemia and hyperkalaemia. He had a cardio-respiratory arrest while receiving the 2^nd^ fluid bolus of 20mls/kg and resuscitation within 3-hours of admission was unsuccessful. | | | | |
| Patient ID. 608 (WD) | The child is a 2 years 3 months old male admitted with a fever, fast and laboured breathing having been referred from a peripheral facility where he was initially seen.  On presentation, his temperature was 37.9^0^C, was severely tachypnoeic (50 breaths/min), tachycardic (169 beats/min), 83% oxygen saturation in room air and MAP 69mmHg. He had all clinical features of WHO shock present (i.e. prolonged capillary refill time >3 seconds, weak & rapid pulse, cool extremities with temperature gradient from toe-to-shin and impaired level of consciousness – prostration).  Investigations at admission: Anaemia (Hb 5.4mg/dL), lactic acidosis (13.0mmol/L), leucocytosis (WBC count 23.2 X 10^6^/L), hypoglycaemia (1.2mmol/L) and a positive rapid diagnostic test for malaria.  Fluid management: He received emergency treatment for the hypoglycaemia and 2 fluid boluses (20mls/kg), then maintenance fluid (4mls/kg/hr). He died 5-hours after admission while awaiting a transfusion (patient’s blood type was not available in the blood bank). | | | | |
| Patient ID. 609 (WM) | The child is a 2 years 1 month old male with a history of fever for 5 days, progressively worsening cough and difficulty in breathing prior to admission and was known to have confirmed sickle cell disease.  On presentation, his temperature was 37.6^0^C, was tachypnoeic (37 breaths/min), heart rate was 136 beats/min, had 85% oxygen saturation in room air and MAP 65mmHg. He had all clinical features of WHO shock present (prolonged capillary refill time >3 seconds, weak & rapid pulse, cool extremities with a temperature gradient from toe-to-shin and impaired level of consciousness – comatose).  Investigations at admission: Severe anaemia (Hb 1.9mg/dL), lactic acidosis (11.9mmol/L), leucocytosis (WBC count 17.1 X 10^6^/L), hyperglycaemia (>11.3mmol/L). Malaria rapid diagnostic test was negative.  Fluid management: The child received insulin for emergency treatment of the hyperglycaemia, was started on maintenance fluid at 4mls/kg/hr while awaiting blood transfusion (he had coarse crackles bilaterally and was not given any 20mls/kg fluid bolus). He died shortly after admission before blood transfusion was administered. | | | | |
| Patient ID. 628 (SK) | The child is a 1 year old female admitted with a 2-day history of cough and fever followed by an acute onset difficulty in breathing preceding the admission.  At presentation to hospital, her temperature was 38.1^0^C, was tachypnoeic (49 breaths/min), heart rate was 192 beats/min, had very low oxygen saturation (32%) in room air but improved on supplemental high-flow oxygen to 93% and a MAP 78mmHg. He had all clinical features of WHO shock present (i.e. prolonged capillary refill time >3 seconds, weak & rapid pulse, cool extremities with a temperature gradient from toe-to-shin and impaired level of consciousness: comatose).  Investigations at admission: Anaemia (Hb 4.7mg/dL), lactic acidosis (13.7mmol/L). Random blood glucose test was normal and malaria rapid diagnostic test was negative.  Fluid management: The child improved after a 20mls/kg bolus of normal saline and was started on maintenance fluid at 4mls/kg/hr. She went into sudden cardiorespiratory arrest 16-hours into admission and resuscitation was unsuccessful. | | | | |
| Patient ID. 629 (OEF) | | The child is a 10 month old male admitted to hospital with an acute onset of fever and abdominal pain accompanying refusal to breastfeed and diarrhoea X1 episode.  At presentation to hospital, he was severely febrile (38.1^0^C body temperature), tachypnoeic (50 breaths/min), tachycardic (221 beats/min). Oxygen saturation was 99% and MAP was 71mmHg. He had all clinical features of WHO shock present (prolonged capillary refill time >3 seconds, weak & rapid pulse, cool extremities with a temperature gradient from toe-to-shin and impaired level of consciousness – prostrate).  Investigations at admission: Anaemia (Hb 2.1mg/dL), lactataemia (3.2mmol/L). Malaria rapid diagnostic test was negative and random blood glucose was 8.9mmol/L.  Fluid management: The child was still tachycardic after receiving 2 fluid boluses of 20mls/kg and died while receiving the third fluid bolus (and awaiting blood for transfusion). | | | |
| Non-WHO shock | |  | | | |
| Patient ID. 604 (ML) | | The child is a 4 year old male presenting to hospital in coma with a history of convulsions but no other co-morbidities.  At admission his body temperature was 36.7^0^C (with history of anti-pyretic treatment), severely tachpnoeic (66 breaths/min), tachycardic (166 beats/min), 96% oxygen saturation and MAP of 71mmHg. On examination, he had cold extremities with toe-to-shin temperature gradient and was comatose but his pulse was bounding and the capillary refill was 2 seconds.  Investigations at admission: Lactataemia (6.4mmol/L), hypoglycaemia (random blood glucose 1.4mmol/L), hyponatraemia (sodium 130mmol/L), leucocytosis (WBC count 15.6 X 10^6^/L). Malaria rapid diagnostic test was positive. Haemoglobin was normal (Hb 13.9mg/dL)  Fluid management: The patient received maintenance fluid (4mls/kg/hr) over 6 hours with good resolution of shock symptoms and was switched to oral rehydration therapy. At 24 hours the lactic acidosis had resolved (lactate 1.0mmol/L). However, the child developed aspiration pneumonia 40-hours into admission following a prolonged seizure episode and resuscitation was unsuccessful. | | | |
| Patient ID. 605 (WB) | | The child is a 1 year 11 month old male with a prolonged history of fever for 9 days and the index episode of passing dark coloured urine on the day of admission. During the presenting illness, the child had been admitted at a local dispensary for 8 days and received two emergency blood transfusions prior to referral on day 9 when he started passing dark urine.  At admission the temperature was 37^0^C, respiratory rate was 38 breaths/min and oxygen saturation was 98% in room air. He was tachycardic (heart rate 160 beats/min) and the MAP was 74mmHg. Physical examination findings: He had impaired level of consciousness – comatose, had a weak & rapid pulse but did not have a toe-to-shin temperature gradient and the capillary refill time was 1 second.  Investigations at admission: Lactate level was raised (3.2mmol/L), leucocytosis (WBC count 47.9 X 10^6^/L). Haemoglobin level was normal (Hb 12.0mg/dL) and malaria rapid diagnostic test was negative. Platelet count was 180 X 10^6^/L at admission.  Fluid management: The patient received maintenance IV fluid at 4mls/kg/hr over four hours and symptoms of shock resolved.  About 8-hours into admission he started developing abdominal distension and bleeding. Haemoglobin check at 8-hours was 9.5mg/dL. The child was not previously known to have any bleeding abnormalities and had not had sickle test done. He progressively deteriorated; maintenance fluid was restarted at 4mls/kg/hr when he developed tachycardia and a rapid & weak pulse. His lactate levels continued to increase to 4mmol/L while Hb dropped to 6.3mg/dL and the platelet count dropped to 143 X 10^6^/L. He went into cardiorespiratory arrest while awaiting blood transfusion and resuscitation was unsuccessful. | | | |
| Patient ID. 615 (KM) | | The child is a 2-year old female admitted to hospital with a 2 day history of fever and progressive onset of difficulty in breathing.  At admission her body temperature was 36.6^0^C, and she had tachypnoea (58 breaths/min), tachycardia (164 beats/min). Oxygen saturation was 98% in room air and the MAP was 59mmHg. Physical examination findings: She had cool peripheries, weak radial pulse and altered level of consciousness but the capillary refill time was 2 seconds.  Investigations at admission: Lactic acidosis (7.9mmol/L), anaemia (4.8mg/dL) and a positive rapid diagnostic test for malaria.  Fluid management: The child received normal saline maintenance fluid at 4mls/kg/hr and blood transfusion (10mls/kg over 3 hours) X2. There was resolution of the acute shock symptoms and the patient was switched to oral rehydration. Despite resolution of shock symptoms, reduction of lactate levels to 2.8mmol/L and clinical improvement over the first 24-hours, the child developed oliguria 32-hours into admission and the creatinine levels increased to 2.6mg/Dl (from 0.7mg/dL at admission). A fluid challenge was attempted but the child went into cardiorespiratory arrest and resuscitation attempts were unsuccessful. | | | |
| All patients received antibiotic therapy, correction of hypoglycaemia as well as supplemental oxygen and anti-malarial treatment (if indicated) based on the standard national Paediatric treatment protocols derived from the WHO guideline | | | | | |

**Table S4:** Echocardiography data at admission by shock criteria. Table showing comparison of baseline echocardiography data among patients who died based on the number of shock criteria present at admission

| **Parameter Medians (IQR)** | **All fatalities**  **(n, 9)** | **≥3 shock criteria**  **(n, 6)** | **≤2 shock criteria**  **(n, 3)** | **U*** | **p-trend*** |
| --- | --- | --- | --- | --- | --- |
| Tei index | 0.12 (0.10-0.36) | 0.11 (0.08-0.12) | 0.36 (0.29-0.53) | 1.43 | 0.20 |
| Ejection Fraction | 59 (47-63) | 61 (50-64) | 53 (49-57) | 0.78 | 0.46 |
| Fractional shortening | 30 (23-32) | 31 (25-33) | 26 (24-28) | 0.91 | 0.39 |
| Stroke Volume (SV)(mls) | 19 (14-24) | 17 (10-23) | 20 (19-22) | 0.44 | 0.67 |
| SV Index (mls/m^2^) | 36 (26-47) | 34 (24-46) | 36 (34-43) | 0.18 | 0.86 |
| End Diastolic Volume (EDV (mls) | 33 (22-45) | 27 (15-50) | 42 (38-44) | 0.17 | 0.86 |
| EDVI (mls/m^2^) | 68 (41-93) | 55 (36-99) | 83 (68-88) | 0.11 | 0.92 |
| SVR (dynes.sec/cm^5^) | 586 (430-791) | 688 (469-888) | 563 (451-673) | 0.49 | 0.64 |
| SVRI (dynes.sec/cm^5^/m^2^) | 1,231 (811-2,077) | 1,684 (931-2,376) | 1,114 (905-1,172) | 1.10 | 0.31 |
| Cardiac Output (mls/min) | 2.9 (1.8-4.1) | 2.4 (1.8-3.8) | 2.9 (2.7-3.6) | 0.39 | 0.71 |
| Cardiac Index (mls/min/m^2^) | 5.4 (4.9-8.4) | 6.0 (5.0-7.9) | 5.0 (4.9-6.8) | 0.16 | 0.88 |
| IVCCI (%),) | 35 (21-40) | 30 (20-39) | 36 (29-40) | 0.17 | 0.87 |
| TAPSE (mm) | 14 (12-15) | 12 (9-14) | 19 (17-19) | 3.14 | 0.02 |
| MAPSE (mm) | 10 (8-10) | 8 (7-11) | 10 (10-10) | 0.85 | 0.42 |
| E’, | 0.11 (0.09-0.15) | 0.11 (0.10-0.13) | 0.15 (0.12-0.16) | 0.64 | 0.54 |
| E/E’ | 7.91 (5.67-9.36) | 7.75 (6.15-9.78) | 7.99 (6.58-8.68) | 0.30 | 0.78 |
| E/A | 1.63 (1.04-1.69) | 1.66 (1.14-2.19) | 1.15 (1.10-1.39) | 0.90 | 0.40 |
| S’ | 0.07 (0.05-0.07) | 0.06 (0.05-0.07) | 0.07 (0.06-0.08) | 0.00 | 1.00 |
| Global Radial Strain | 23 (23-40) | 23 (17-29) | 41 (32-42) | 2.42 | 0.05 |
| Global Circumferential Strain | -21 (-24) - (-13) | -17 (-25) – (-9) | -21 (-23) – (-17) | 0.28 | 0.79 |
| Global Longitudinal Strain | -13 (-17) - (-13) | -13 (-15) – (-9) | -17 (-19) – (-15) | 2.08 | 0.07 |

*Mann Whitney, U-statistic and p-value

EF, ejection fraction; FS, fractional shortening; SV, stroke volume; SVI, stroke volume index; EDV, end-diastolic volume; EDVI, end-diastolic volume index; SVR, systemic vascular resistance; SVRI, systemic vascular resistance index; CO, cardiac output; CI, cardiac index; IVCCI, inferior vena cava collapsibility index; TAPSE, tricuspid annular plane systolic excursion; MAPSE, mitral annular plane systolic excursion; E, mitral inflow; E’, early diastolic mitral inflow; E/A, early to late mitral diastolic filling ratio; S’, systolic left ventricular peak velocity; GRS, global radial strain; GCS, global circumferential strain; GLS, global longitudinal strain.

**Table S5:** Echocardiography data over time. Table showing trend of echocardiography parameters at admission, post-fluid resuscitation, at 24-hours and day-28

| **n (%) or Medians (IQR)** | **Pre-fluid**  **(n, 30)** | **Post-fluid**  **(n, 25)** | **24 hours**  **(n, 23)** | **Day 28**  **(n, 21)** | **F** | **p-trend*** |
| --- | --- | --- | --- | --- | --- | --- |
| **Global Function** |  |  |  |  |  |  |
| Tei index | 0.15 (0.10 - 0.23) | 0.15 (0.07 - 0.27) | 0.22 (0.14 - 0.35) | 0.19 (0.11 - 0.37) | 1.08 | 0.36 |
| Low Tei index, n (%) | 24 (80) | 15 (60) | 12 (52) | 10 (48) |  |  |
| Global Radial Strain, GRS | 36 (23 - 43) | 25 (21 - 38) | 26 (20 - 35) | 33 (25 - 46) | 1.99 | 0.16 |
| Reduced GRS, n (%) | 25 (83) | 19 (76) | 15 (65) | 6 (29) |  |  |
| Global Circumferential Strain, GCS | -19 (-23) - (-15) | -17 (-22) - (-14) | -17 (-23) - (-14) | -20 (-21) - (-15) | 0.01 | 0.90 |
| Reduced GCS, n (%) | 14 (47) | 10 (40) | 9 (39) | 5 (24) |  |  |
| Global Longitudinal Strain | -20 (-23) - (-16) | -22 (-24) - (-18) | -22 (-23) - (-17) | -19 (-23) - (-19) | 0.57 | 0.45 |
| Reduced GLS, n (%) | 7 (23) | 5 (20) | 3 (13) | 2 (10) |  |  |
| **Systolic function** |  |  |  |  |  |  |
| Ejection Fraction (EF) | 60 (54 - 64) | 61 (58 - 66) | 64 (60 - 69) | 63 (60 - 66) | 5.18 | 0.05 |
| Reduced EF, n (%) | 8 (27) | 4 (16) | 2 (9) | 0 (0) |  |  |
| Fractional Shortening (FS) | 30 (27 - 33) | 32 (29 - 35) | 34 (31 - 38) | 33 (31 - 35) | 4.99 | 0.05 |
| Reduced FS, n (%) | 9 (30) | 4 (16) | 2 (9) | 1 (5) |  |  |
| TAPSE (mm), | 16 (14 - 18) | 16 (15 - 19) | 16 (15 - 17) | 18 (16 - 19) | 1.54 | 0.22 |
| Reduced TAPSE, n (%) | 10 (33) | 4 (16) | 6 (26) | 3 (14) |  |  |
| MAPSE (mm) | 11 (10 - 12) | 11 (10 - 12) | 11 (10 - 12) | 11 (10 - 12) | 0.02 | 0.99 |
| Reduced MAPSE, n (%) | 7 (23) | 5 (20) | 4 (17) | 4 (19) |  |  |
| **Diastolic function** |  |  |  |  |  |  |
| E’ | 0.15 (0.12 - 0.18) | 0.16 (0.14 - 0.17) | 0.13 (0.13 - 0.16) | 0.14 (0.12 - 0.15) | 1.66 | 0.20 |
| E/E’ | 7.95 (5.92 - 9.36) | 7.70 (6.53 - 8.78) | 8.57 (6.30 - 9.76) | 7.44 (6.05 - 9.30) | 0.02 | 0.90 |
| Low E/E’, n (%) | 15 (50) | 13 (52) | 10 (44) | 6 (29) |  |  |
| E/A | 1.24 (1.12 - 1.63) | 1.33 (1.24 - 1.97) | 1.35 (1.26 - 1.50) | 1.60 (1.32 - 1.85) | 1.15 | 0.29 |
| S’ | 0.08 (0.06 - 0.09) | 0.07 (0.07 - 0.08) | 0.08 (0.07 - 0.09) | 0.07 (0.06 - 0.07) | 1.28 | 0.26 |
| **Hemodynamics** |  |  |  |  |  |  |
| Stroke Volume (mL), | 19 (16 - 23) | 23 (17 - 24) | 24 (21 - 27) | 21 (18 - 25) | 2.30 | 0.13 |
| Stroke Volume Index, SVI (mL/m^2^) | 39 (32 - 42) | 42 (37 - 46) | 47 (41 - 49) | 43 (37 - 47) | 4.98 | 0.04 |
| Low SVI, n (%) | 21 (70) | 15 (60) | 8 (35) | 8 (38) |  |  |
| High SVI, n (%) | 2 (7) | 3 (12) | 5 (22) | 3 (14) |  |  |
| Cardiac Output (mL/min) | 3.0 (2.4 - 3.7) | 2.9 (2.4 - 3.9) | 3.1 (2.8 - 3.6) | 2.6 (2.3 - 2.7) | 3.78 | 0.06 |
| Cardiac Index (mL/min/m^2^ | 6.0 (4.9 - 7.2) | 5.8 (5.2 - 7.0) | 6.1 (5.6 - 7.3) | 4.8 (4.6 - 6.0) | 3.91 | 0.06 |
| End Diastolic Volume (mL) | 31 (26 - 40) | 34 (28 - 39) | 35 (32 - 40) | 34 (30 - 41) | 0.23 | 0.64 |
| EDV Index, EDVI (mL/m^2^) | 64 (52 - 71) | 64 (58 - 72) | 68 (64 - 79) | 67 (62 - 75) | 0.49 | 0.49 |
| Low EDVI, n (%) | 12 (40) | 11 (44) | 4 (17) | 4 (19) |  |  |
| Systemic Vascular Resistance (SVR) (dynes.sec/cm^5^) | 432 (395 - 574) | 467 (391 - 504) | 441 (351 - 503) | 461 (397 - 506) | 3.14 | 0.08 |
| SVR Index (dynes.sec/cm^5^/m^2^) | 884 (764 - 1,213) | 957 (750 - 1,086) | 866 (597 - 1,039) | 898 (713 - 1,038) | 3.06 | 0.08 |
| Low SVRI, n (%) | 15 (50) | 13 (52) | 12 (52) | 8 (38) |  |  |
| High SVRI, n (%) | 5 (17) | 1 (4) | 1 (4) | 1 (5) |  |  |
| Low cardiac index, n (%) | 3 (10) | 1 (4) | 1 (4) | 0 (0) |  |  |
| IVCCI | 28 (19 - 40) | 29 (18 - 33) | 23 (19 - 34) | 29 (14 - 33) | 0.59 | 0.45 |
| Low IVCCI, n (%) | 17 (57) | 16 (64) | 15 (65) | 9 (43) |  |  |

*F-statistic and p-value of trend test from baseline to 1-month follow-up

EF, ejection fraction; FS, fractional shortening; SV, stroke volume; SVI, stroke volume index; EDV, end-diastolic volume; EDVI, end-diastolic volume index; SVR, systemic vascular resistance; SVRI, systemic vascular resistance index; CO, cardiac output; CI, cardiac index; IVCCI, inferior vena cava collapsibility index; TAPSE, tricuspid annular plane systolic excursion; MAPSE, mitral annular plane systolic excursion; E, mitral inflow; E’, early diastolic mitral inflow; E/A, early to late mitral diastolic filling ratio; S’, systolic left ventricular peak velocity; GRS, global radial strain; GCS, global circumferential strain; GLS, global longitudinal strain.

**Table S6:** Baseline electrocardiography variables at admission for all patients; survivors and fatalities

|  | **All**  **(n, 30)** | **Survivors**  **(n, 21)** | **Fatalities**  **(n, 9)** | ***U**** | **p-value*** |
| --- | --- | --- | --- | --- | --- |
| PR-interval (milliseconds), median (IQR) | 174 (106 – 186) | 176 (117-191) | 112 (105-148) | 1.85 | 0.08 |
| QRS-duration (milliseconds), median (IQR) | 68 (60 – 72) | 69 (62-72) | 68 (62-73) | 0.54 | 0.60 |
| QT-duration, (milliseconds), median (IQR) | 254 (248 – 278) | 254 (246-275) | 258 (249-271) | 0.18 | 0.86 |
| Corrected QT-duration (milliseconds), median (IQR) | 422 (405 – 427) | 423 (409-430) | 414 (403-424) | 1.08 | 0.29 |
| P-wave axis (degrees), median (IQR) | 65 (45 – 70) | 45 (40-65) | 70 (66-71) | 1.62 | 0.07 |
| QRS-complex axis, (degrees), median (IQR) | 60 (35 – 75) | 55 (34-70) | 75 (60-85) | 1.76 | 0.09 |
| T-wave axis, (degrees), median (IQR) | 55 (35 – 60) | 50 (35-56) | 60 (53-63) | 0.19 | 0.85 |
| P duration, (milliseconds), median (IQR) | 146 (80 – 160) | 154 (87-165) | 82 (77-120) | 1.98 | 0.06 |
| RR-interval, (milliseconds), median (IQR) | 378 (354 – 424) | 375 (353-417) | 398 (368-436) | 0.80 | 0.43 |
| QT-dispersion, (milliseconds), median (IQR) | 30 (22 – 48) | 28 (22-41) | 38 (29-44) | 0.68 | 0.50 |
| Corrected QT-dispersion, (milliseconds), median (IQR) | 46 (36 – 78) | 45 (35-68) | 60 (43-73) | 0.59 | 0.56 |

* Test statistic: Mann Whitney *U*-test and p-value comparing baseline characteristics of survivors versus fatalities

**Table S7:** Electrocardiography data at admission by shock criteria. Table showing comparison of baseline electrocardiography data among patients who died based on the number of shock criteria present at admission

| **Parameter median (IQR)** | **Fatalities**  **(n, 9)** | **≥3 criteria**  **(n, 6)** | **≤2 criteria**  **(n, 3)** | **U*** | **p*** |
| --- | --- | --- | --- | --- | --- |
| PR-interval (milliseconds) | 112 (105-148) | 108 (98-131) | 112 (110-148) | 0.47 | 0.66 |
| QRS-duration (milliseconds) | 68 (62-73) | 70 (63-80) | 68 (62-69) | 0.88 | 0.41 |
| QT-duration, (milliseconds) | 258 (249-271) | 254 (250-260) | 278 (263-290) | 1.88 | 0.10 |
| Corrected QT-duration (milliseconds) | 414 (403-424) | 404 (394-416) | 424 (418-426) | 1.58 | 0.16 |
| P-wave axis (degrees) | 70 (66-71) | 73 (71-74) | 63 (59-66) | 2.21 | 0.06 |
| QRS-complex axis, (degrees) | 75 (60-85) | 78 (64-91) | 60 (60-75) | 0.33 | 0.75 |
| T-wave axis, (degrees) | 60 (53-63) | 58 (28-60) | 65 (58-68) | 0.94 | 0.38 |
| P duration, (milliseconds) | 82 (77-120) | 78 (69-101) | 84 (82-120) | 0.48 | 0.65 |
| RR-interval, (milliseconds) | 398 (368-436) | 385 (370-411) | 424 (383-482) | 0.89 | 0.40 |
| QT-dispersion, (milliseconds) | 38 (29-44) | 44 (37-51) | 30 (24-34) | 1.76 | 0.12 |
| Corrected QT-dispersion, (milliseconds) | 60 (43-73) | 70 (56-84) | 41 (35-53) | 1.65 | 0.14 |

*Mann Whitney, U-statistic and p-value

**Table S8:** Electrocardiography data over time. Table showing trend of electrocardiography parameters at admission, 24-hours and day-28

| **n (%) or Medians (IQR)** | **Admission**  **(n, 30)** | **24 hours**  **(n, 23)** | **Day 28**  **(n, 21)** | **F** | **p-trend*** |
| --- | --- | --- | --- | --- | --- |
| PR-interval (milliseconds) | 174 (107 - 185) | 120 (107 - 156) | 122 (104 - 140) | 0.16 | 0.69 |
| Prolonged PR-interval, n (%) | 15 (50) | 7 (30) | 6 (29) |  |  |
| QRS-duration (milliseconds) | 68 (61 - 72) | 72 (69 - 74) | 68 (66 - 70) | 0.07 | 0.79 |
| Prolonged QRS-duration, n (%) | 3 (10) | 5 (17) | 3 (14) |  |  |
| QT-duration, (milliseconds) | 254 (248 - 276) | 294 (264 - 313) | 306 (284 - 336) | 27.19 | <0.001 |
| Corrected QT-duration (milliseconds) | 422 (408 - 427) | 435 (426 - 453) | 435 (425 - 449) | 3.20 | 0.08 |
| Prolonged corrected QT- duration, n (%) | 1 (3) | 10 (44) | 7 (33) |  |  |
| P-wave axis (degrees) | 65 (45 - 75) | 60 (50 - 60) | 50 (33 - 55) | 3.62 | 0.06 |
| Abnormal P-wave axis, n (%) | 0 (0) | 0 (0) | 0 (0) |  |  |
| QRS-complex axis, (degrees) | 60 (40 - 75) | 55 (25 - 70) | 40 (20 - 70) | 2.20 | 0.14 |
| Right deviation, n (%) | 2 (7) | 2 (9) | 0 (0) |  |  |
| Left deviation, n (%) | 0 (0) | 0 (0) | 0 (0) |  |  |
| T-wave axis, (degrees) | 55 (40 - 60) | 50 (40 - 63) | 45 (35 - 50) | 0.0001 | 0.99 |
| Abnormal T-wave axis, n (%) | 3 (10) | 0 (0) | 0 (0) |  |  |
| P duration, (milliseconds) | 146 (81 - 160) | 88 (83 - 116) | 84 (76 - 96) | 7.35 | 0.009 |
| Prolonged P-duration, n (%) | 15 (50) | 6 (26) | 2 (10) |  |  |
| RR-interval, (milliseconds) | 378 (357 - 421) | 422 (394 - 476) | 502 (440 - 578) | 22.61 | <0.0001 |
| Prolonged RR-interval, n (%) | 8 (27) | 14 (61) | 13 (62) |  |  |
| QT-dispersion, (milliseconds) | 30 (23 - 44) | 50 (31 - 64) | 30 (20 - 40) | 0.03 | 0.87 |
| Corrected QT-dispersion, (milliseconds) | 46 (37 - 72) | 69 (46 - 102) | 39 (28 - 56) | 0.82 | 0.37 |
| Prolonged corrected QT-dispersion, n (%) | 4 (13) | 6 (26) | 0 (0) |  |  |

*F and p-value of trend test from baseline to 1-month follow-up

**Table S9:** Baseline cardiac and microvascular biomarker data. Table showing comparison of baseline cardiac and microvascular biomarkers based on outcome status

| **Parameter Medians (IQR)** | **All**  **(n, 30)** | **Survivors**  **(n, 21)** | **Non-survivors**  **(n, 9)** | **U*** | **p-*** |
| --- | --- | --- | --- | --- | --- |
| Troponin (ng/mL) | 0.56 (0.05 – 1.33) | 0.07 (0 – 0.96) | 0.26 (0.07 – 0.51) | 1.62 | 0.32 |
| BNP (pg/mL) | 512 (337 – 712) | 520 (328 – 649) | 442 (344 – 664) | 0.46 | 0.88 |
| ANP (pg/mL) | 58 (35.9 – 71.6) | 39 (6.1 – 57.5) | 70 (66.6 – 74.2) | 2.37 | 0.08 |
| Plasma hyaluronan (ng/mL) | 105 (46 – 168) | 68 (45 – 166) | 168 (116 – 168) | 2.06 | 0.15 |
| Urine hyaluronan (ng/mL) | 165 (135 – 168) | 155 (131 – 162) | 168 (168 – 168) | 1.01 | 0.55 |

*Mann Whitney, U-statistic and p-value

**Table S10**: Biomarker profile at admission by shock criteria. Table showing comparison of baseline cardiac and microvascular biomarkers among patients who died based on the number of shock criteria present at admission

| **Parameter Medians (IQR)** | **All fatalities**  **(n, 9)** | **≥3 criteria**  **(n, 6)** | **≤2 criteria**  **(n, 3)** | **U*** | **p*** |
| --- | --- | --- | --- | --- | --- |
| Troponin (ng/mL) | 0.26 (0.07 – 0.51) | 0.67 (0.61 – 0.74) | 0.09 (0.05 – 0.20) | 3.58 | **0.02** |
| BNP (pg/mL) | 442 (344 – 664) | 798 (563 – 1,033) | 442 (359 – 567) | 0.99 | 0.38 |
| ANP (pg/mL) | 70 (66.6 – 74.2) | 111 (91 – 130) | 67 (64 – 70) | 16.5 | **<0.01** |
| Plasma hyaluronan (ng/mL) | 168 (116 – 168) | 168 (168 – 168) | 98 (87 – 133) | 0.75 | 0.49 |
| Urine hyaluronan (ng/mL) | 168 (168 – 168) | 168 (168 – 168) | 168 (135 – 168) | 1.20 | 0.29 |

*Mann Whitney, U-statistic and p-value

**Table S11**: Biomarker profiles over time. Table showing trend of biomarkers at admission, 8-hours and 24-hours

| **n (%) or Medians (IQR)** | **Baseline: 0 hour**  **(n, 30)** | **8 hours**  **(n, 25)** | **24 hours**  **(n, 23)** | **F*** | **p-trend*** |
| --- | --- | --- | --- | --- | --- |
| Troponin (ng/mL) | 0.11 (0 - 0.90) | 0.01 (0 - 1.26) | 0.00 (0 - 0) | 2.01 | 0.14 |
| High troponin**, n (%) | 16 (53) | 9 (36) | 2 (9) |  |  |
| Beta-Brain Natriuretic Peptide (pg/mL) | 518 (332 - 673) | 457 (270 - 624) | 482 (269 - 632) | 0.89 | 0.42 |
| High BNP**, n (%) | 21 (70) | 15 (60) | 7 (30) |  |  |
| Alpha-Atrial Natriuretic Peptide (pg/mL) | 53 (20 - 70) | 1.58 (0.04-34.90) | 0.39 (0 - 1.36) | 11.78 | <0.0001 |
| High ANP**, n (%) | 10 (33) | 1 (4) | 0 (0) |  |  |
| Plasma hyaluronan (ng/mL) | 91 (46 - 168) | 163 (66 - 168) | 132 (67 - 168) | 1.37 | 0.26 |
| High plasma hyaluronan**, n (%) | 12 (40) | 15 (60) | 7 (30) |  |  |
| Urine hyaluronan (ng/mL) | 165 (137 - 168) | - | 148 (127 - 168) | 2.2 | 0.15 |

*F and p-value of trend test from baseline to 1-month follow-up

Reference values for pediatric parameters (**no pediatric reference)

Troponin >0.1 µg/L ^17^

β-BNP >300 pg/ml ^18^

α-ANP >60pg/ml ^19,20^

**Hyaluronan >100 ng/mL (in plasma) and >300 ng/mL (in urine) ^22^ .

**Table S12:** Odds of death outcome based on admission variables. Univariate analysis showing odds of death for deranged admission variables

| **Variables** | **Odds ratio** | **95% CI** | **p-value** |
| --- | --- | --- | --- |
| **Clinical** |  |  |  |
| Temperature (36^0^C < temp > 39^0^C) | 1.66 | 0.78 - 3.51 | 0.19 |
| Tachypnoea* | 0.99 | 0.94 - 1.07 | 0.96 |
| Tachycardia^╬^ | 1.01 | 0.99 - 1.05 | 0.12 |
| Hypoxia (oxygen saturations <90%) | 1.12 | 0.97 - 1.30 | 0.13 |
| Prolonged capillary refill time (>3 seconds) | 1.22 | 0.17 - 1.67 | 0.29 |
| **Laboratory** |  |  |  |
| Hyponatremia (sodium <125 mmol/L) | 1.19 | 0.94 - 1.51 | 0.14 |
| Hyperkalemia (potassium >5.5 mmol/L) | 1.33 | 1.09 - 1.89 | **0.04** |
| Hypercreatininemia (Creatinine >80µmol/L) | 0.99 | 0.97 - 1.01 | 0.26 |
| Hyperlactatemia (Lactate >4.0 mmol/L) | 1.87 | 1.04 - 2.45 | **0.03** |
| Hypoglycemia (Glucose <3.0 mmol/L) | 2.07 | 1.08 - 3.95 | **0.03** |
| Thrombocytopenia (Platelets <160 X 10^9^/L) | 1.00 | 0.99 - 1.01 | 0.55 |
| Leukocytosis^+^ | 0.97 | 0.92 - 1.02 | 0.27 |
| Severe anemia (Hemoglobin <5 g/dL) | 1.73 | 1.42 - 7.20 | **0.04** |
| **Cardiac biomarkers** |  |  |  |
| High troponin (>0.1 µg/mL) | 4.01 | 0.63 - 25.57 | 0.08 |
| High alpha-atrial natriuretic peptide (>60 pg/mL) | 0.96 | 0.91 - 1.00 | 0.19 |
| High beta-brain natriuretic peptide (>300 pg/mL) | 1.00 | 0.99 - 1.00 | 0.63 |
| High plasma hyaluronan (>100 ng/mL) | 0.98 | 0.96 - 1.00 | 0.07 |
| High urine hyaluronan (>300 ng/mL) | 0.97 | 0.92 - 1.03 | 0.32 |
| **Electrocardiography^** |  |  |  |
| Prolonged PR-interval | 1.02 | 0.99 - 1.04 | 0.12 |
| Prolonged QRS duration | 0.97 | 0.87 - 1.07 | 0.51 |
| Prolonged corrected QT-time | 1.03 | 0.97 - 1.08 | 0.34 |
| QRS-complex axis deviation | 0.97 | 0.94 - 1.01 | 0.13 |
| Abnormal T-wave axis | 0.99 | 0.97 - 1.02 | 0.89 |
| Prolonged P-duration | 1.01 | 0.99 - 1.04 | 0.10 |
| Prolonged RR-interval | 0.99 | 0.98 - 1.01 | 0.48 |
| Prolonged corrected QT-dispersion time | 0.99 | 0.96 - 1.02 | 0.58 |
| **Echocardiography^^** |  |  |  |
| Low Tei index | 0.03 | 0.0001 – 5.70 | 0.19 |
| Reduced Global Radial Strain | 0.97 | 0.91 - 1.03 | 0.29 |
| Reduced Global Circumferential Strain | 0.76 | 0.60 - 0.95 | **0.02** |
| Reduced Global Longitudinal Strain | 0.82 | 0.69 - 0.99 | **0.04** |
| Reduced ejection fraction | 1.04 | 0.96 - 1.12 | 0.39 |
| Reduced fractional shortening | 1.13 | 0.96 - 1.34 | 0.15 |
| Reduced TAPSE | 1.41 | 1.02 - 1.95 | **0.04** |
| Reduced MAPSE | 1.95 | 1.09 - 3.48 | **0.02** |
| Low E/E’ ratio | 0.95 | 0.67 - 1.37 | 0.80 |
| Low stroke volume index | 1.01 | 0.95 - 1.08 | 0.68 |
| Low end-diastolic volume index | 0.98 | 0.95 - 1.02 | 0.29 |
| Systemic vascular resistance index (900 <SVRI > 1440 dynes.sec/cm^5^/m^2^) | 0.99 | 0.99 - 1.01 | 0.61 |
| Low cardiac index | 0.94 | 0.61 - 1.45 | 0.78 |
| Low inferior vena cava collapsibility index | 0.99 | 0.94 - 1.04 | 0.65 |

*Tachypnoea criteria: >34 brpm in children < 12 months; >22 brpm in >1 to 5yrs of age; and >18 brpm in > 5 yrs

^╬^Tachycardia criteria: >180 bpm in < 12 months; >160 bpm in > 1 to 5 yrs; and >140 bpm in > 5 yrs

^+^Leukocytosis criteria: White blood cell count of >17.5 X 10^9^/L in in children < 12 months of age; >15.5 X 10^9^/L in in >1 to 5 yrs of age; and >13.5 X 10^9^/L in in > 5 yrs

^Based on published electrocardiogram references for age^1,23^

^^Based on published echocardiogram references for age^4,24^

# SUPPLEMENTARY FIGURES

**Figure S1** MAPS study flow diagram.

A total of 54 eligible patients were screened, 24 of whom were excluded for various reasons (outlined) and 30 were included in the final analysis.


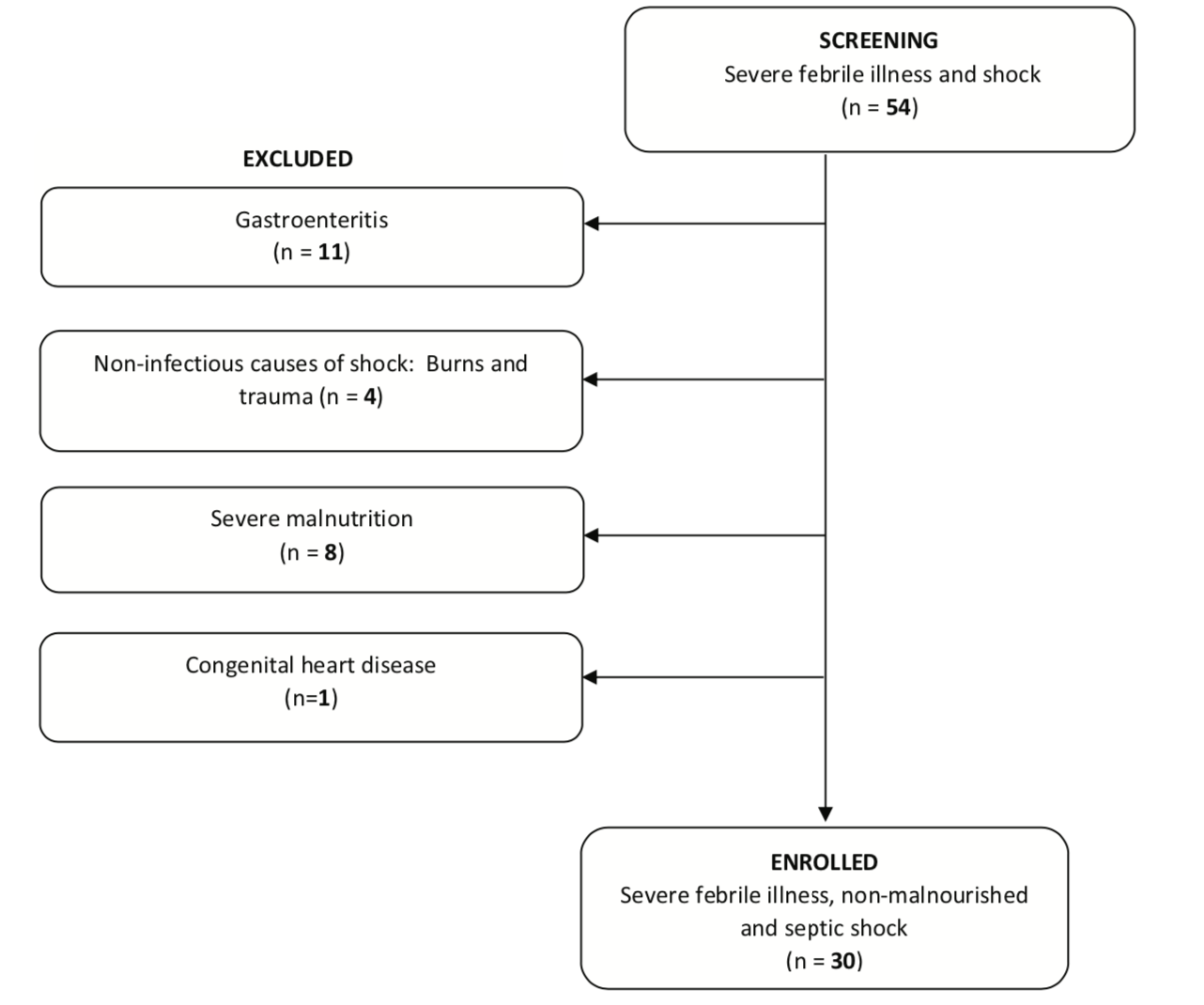


#

### Figure S2 Kaplan-Meier survival estimates at 48-hours for children with and without WHO shock

**Figure S3: Dot-plots showing echocardiographic parameters at admission, post-resuscitation and at 24-hours in survivors (blue) and fatalities (red)**

**(a) Ejection fraction**


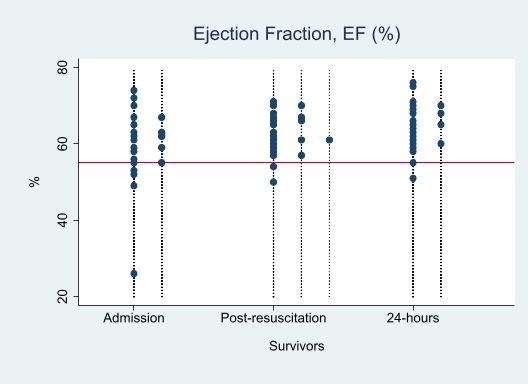

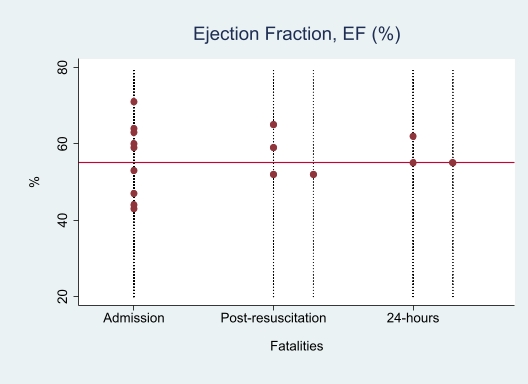


**(b) Cardiac Index (CI)**


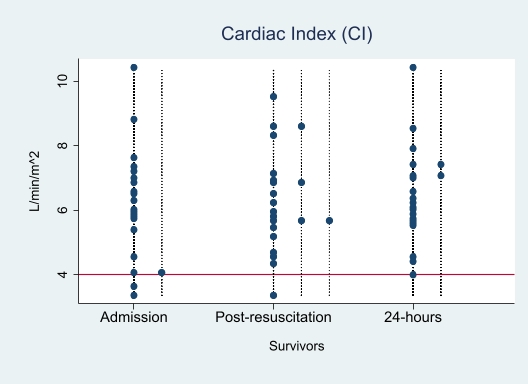

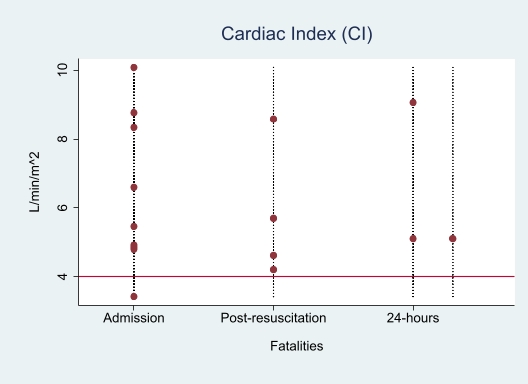


**(c) Systemic vascular resistance index (SVRI)**


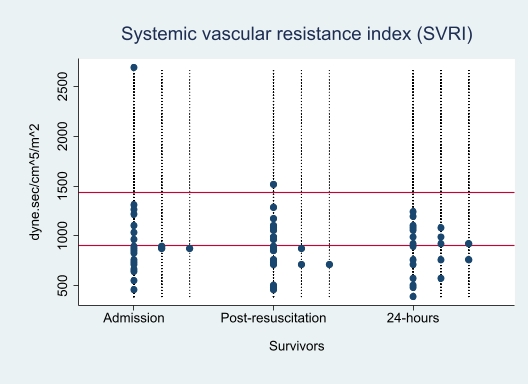

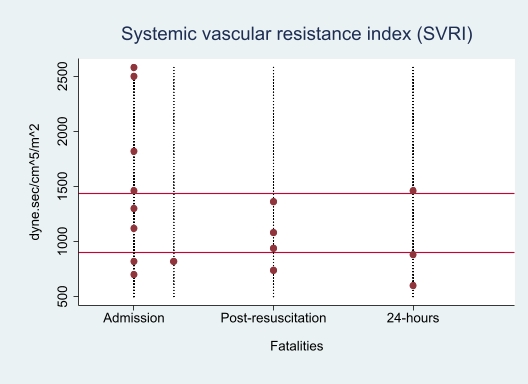


**(d) Global Radial Strain, GRS (%)**


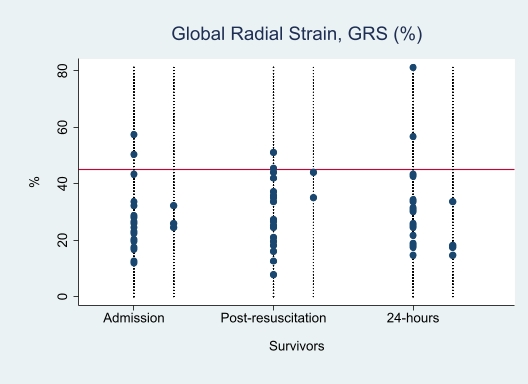

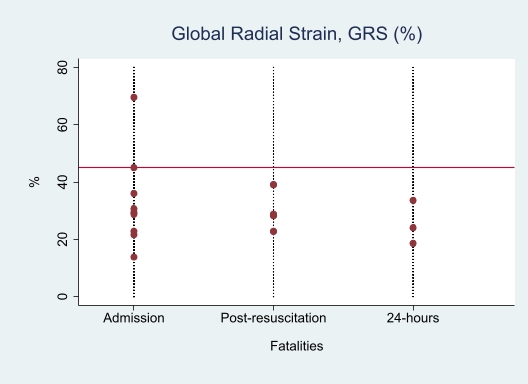


**(e) Global Circumferential Strain, GCS (%)**


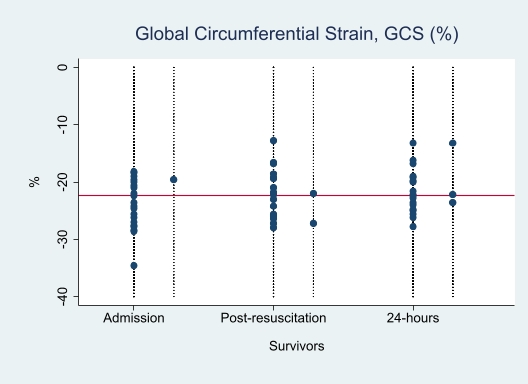

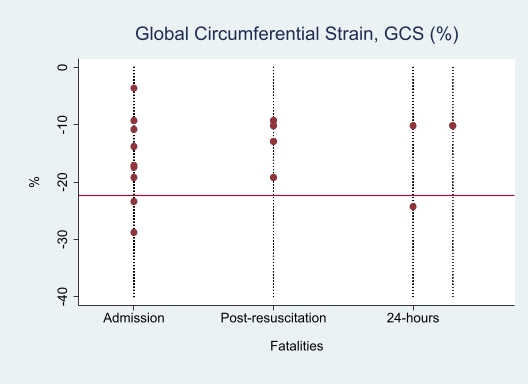


**(f) Global Longitudinal Strain, GLS (%)**


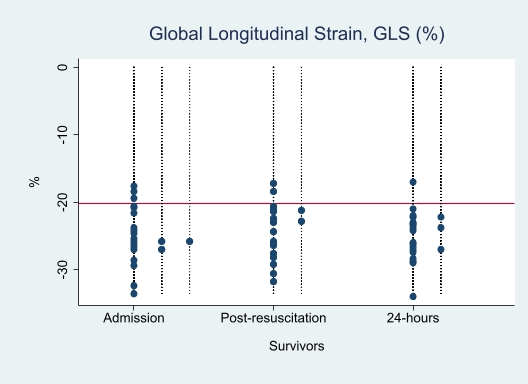

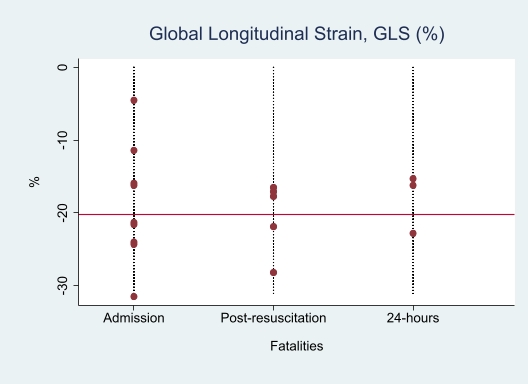


**Figure 3: Dot-plots showing biomarker profiles at admission, post-resuscitation and at 24-hours in survivors (blue) and fatalities (red)**

**(a) Cardiac troponin I (cTnI)**


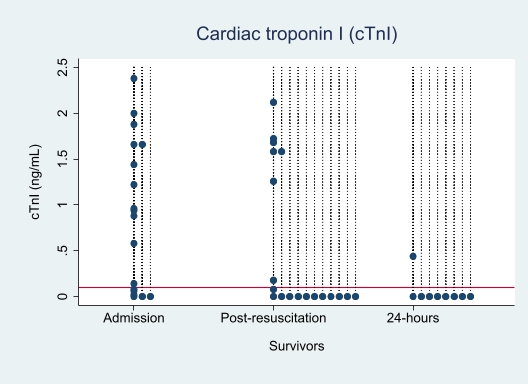

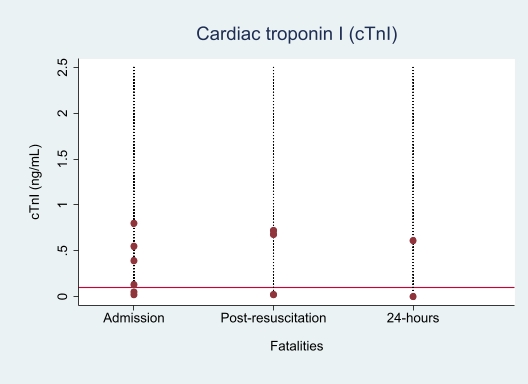


**(b) Alpha-atrial natriuretic peptide (ANP)**


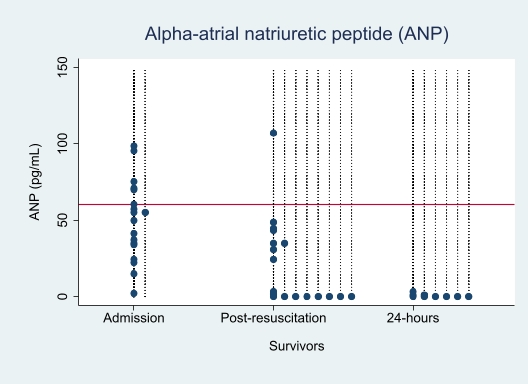

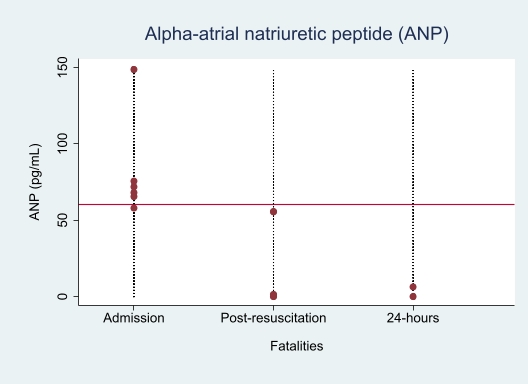


**(c) Beta-brain natriuretic peptide (BNP)**


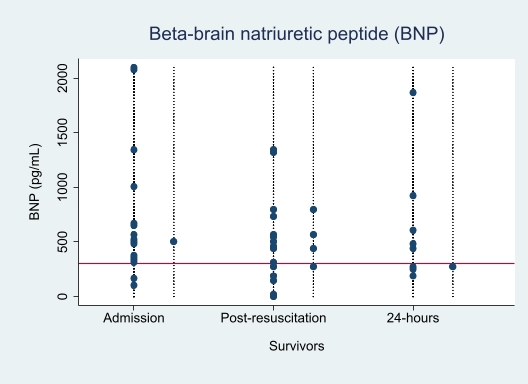

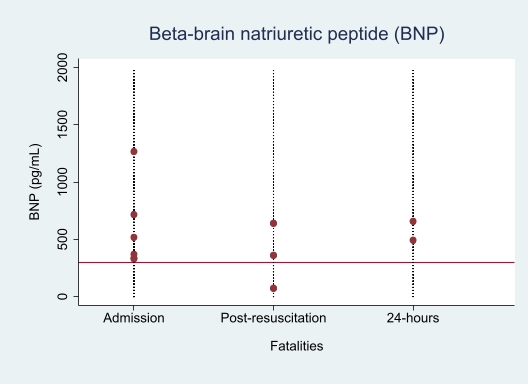


**(d) Hyaluronan in plasma**


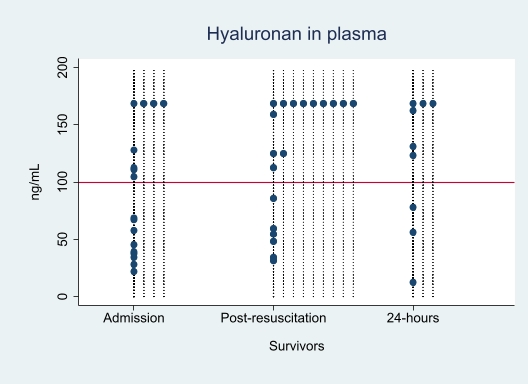

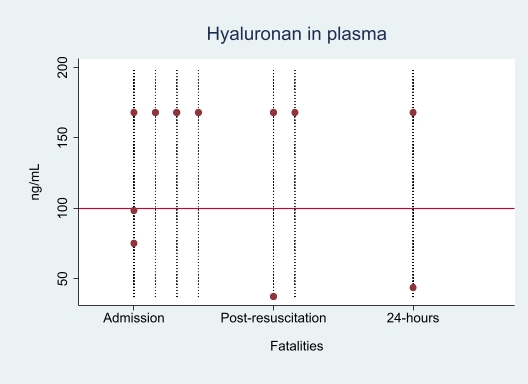


**(e) Hyaluronan in urine**


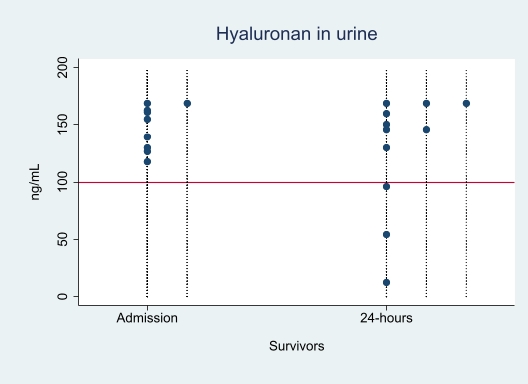

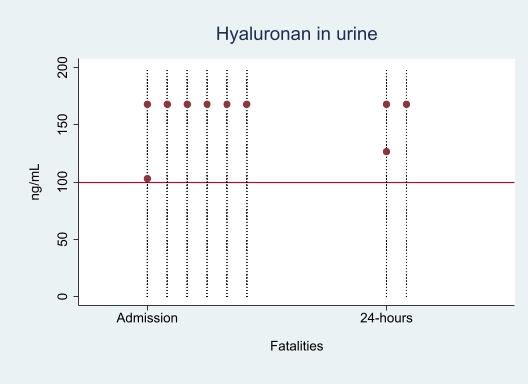


# REFERENCES

1. Kligfield P, Gettes LS, Bailey JJ, et al. Recommendations for the standardization and interpretation of the electrocardiogram: part I: The electrocardiogram and its technology: a scientific statement from the American Heart Association Electrocardiography and Arrhythmias Committee, Council on Clinical Cardiology; the American College of Cardiology Foundation; and the Heart Rhythm Society: endorsed by the International Society for Computerized Electrocardiology. *Circulation.* 2007;115(10):1306-1324.

2. Bazett HC. An analysis of the time-relations of electrocardiograms. *Heart-J Stud Circ.* 1920;7(4):353-370.

3. Tutar HE, Ocal B, Imamoglu A, Atalay S. Dispersion of QT and QTc interval in healthy children, and effects of sinus arrhythmia on QT dispersion. *Heart.* 1998;80(1):77-79.

4. Lopez L, Colan SD, Frommelt PC, et al. Recommendations for quantification methods during the performance of a pediatric echocardiogram: a report from the Pediatric Measurements Writing Group of the American Society of Echocardiography Pediatric and Congenital Heart Disease Council. *J Am Soc Echocardiogr.* 2010;23(5):465-495; quiz 576-467.

5. Haycock GB, Schwartz GJ, Wisotsky DH. Geometric method for measuring body surface area: a height-weight formula validated in infants, children, and adults. *J Pediatr.* 1978;93(1):62-66.

6. Tei C, Ling LH, Hodge DO, et al. New index of combined systolic and diastolic myocardial performance: a simple and reproducible measure of cardiac function--a study in normals and dilated cardiomyopathy. *J Cardiol.* 1995;26(6):357-366.

7. Tei C. New non-invasive index for combined systolic and diastolic ventricular function. *J Cardiol.* 1995;26(2):135-136.

8. Karatzis EN, Giannakopoulou AT, Papadakis JE, Karazachos AV, Nearchou NS. Myocardial performance index (Tei index): evaluating its application to myocardial infarction. *Hellenic journal of cardiology : HJC = Hellenike kardiologike epitheorese.* 2009;50(1):60-65.

9. Karaye KM. Relationship between Tei Index and left ventricular geometric patterns in a hypertensive population: a cross-sectional study. *Cardiovasc Ultrasound.* 2011;9:21.

10. Mercier JC, DiSessa TG, Jarmakani JM, et al. Two-dimensional echocardiographic assessment of left ventricular volumes and ejection fraction in children. *Circulation.* 1982;65(5):962-969.

11. Wyatt HL, Heng MK, Meerbaum S, et al. Cross-sectional echocardiography. II. Analysis of mathematic models for quantifying volume of the formalin-fixed left ventricle. *Circulation.* 1980;61(6):1119-1125.

12. Wyatt HL, Meerbaum S, Heng MK, Gueret P, Corday E. Cross-sectional echocardiography. III. Analysis of mathematic models for quantifying volume of symmetric and asymmetric left ventricles. *Am Heart J.* 1980;100(6 Pt 1):821-828.

13. Gorcsan J, 3rd, Tanaka H. Echocardiographic assessment of myocardial strain. *J Am Coll Cardiol.* 2011;58(14):1401-1413.

14. Lang RM, Badano LP, Mor-Avi V, et al. Recommendations for cardiac chamber quantification by echocardiography in adults: an update from the American Society of Echocardiography and the European Association of Cardiovascular Imaging. *J Am Soc Echocardiogr.* 2015;28(1):1-39 e14.

15. Cattermole GN, Leung PY, Mak PS, Chan SS, Graham CA, Rainer TH. The normal ranges of cardiovascular parameters in children measured using the Ultrasonic Cardiac Output Monitor. *Critical care medicine.* 2010;38(9):1875-1881.

16. Pettersen MD, Du W, Skeens ME, Humes RA. Regression equations for calculation of z scores of cardiac structures in a large cohort of healthy infants, children, and adolescents: an echocardiographic study. *Journal of the American Society of Echocardiography : official publication of the American Society of Echocardiography.* 2008;21(8):922-934.

17. Soldin SJ, Murthy JN, Agarwalla PK, Ojeifo O, Chea J. Pediatric reference ranges for creatine kinase, CKMB, Troponin I, iron, and cortisol. *Clin Biochem.* 1999;32(1):77-80.

18. Price JF, Thomas AK, Grenier M, et al. B-type natriuretic peptide predicts adverse cardiovascular events in pediatric outpatients with chronic left ventricular systolic dysfunction. *Circulation.* 2006;114(10):1063-1069.

19. Gangnus T, Burckhardt BB. Potential and Limitations of Atrial Natriuretic Peptide as Biomarker in Pediatric Heart Failure-A Comparative Review. *Front Pediatr.* 2018;6:420.

20. Kotby AA, Taman KH, Sedky HT, Habeeb NM, El-Hadidi ES, Yosseif HS. Atrial natriuretic peptide as a marker of heart failure in children with left ventricular volume overload. *J Paediatr Child Health.* 2013;49(1):43-47.

21. Toyoda H, Muraki F, Imanari T, Kinoshita-Toyoda A. Microdetermination of hyaluronan in human plasma by high-performance liquid chromatography with a graphitized carbon column and postcolumn fluorometric detection. *J Chromatogr B Analyt Technol Biomed Life Sci.* 2011;879(13-14):950-954.

22. Laurent UB, Tengblad A. Determination of hyaluronate in biological samples by a specific radioassay technique. *Anal Biochem.* 1980;109(2):386-394.

23. Dickinson DF. The normal ECG in childhood and adolescence. *Heart.* 2005;91(12):1626-1630.

24. Majonga ED, Rehman AM, McHugh G, et al. Echocardiographic reference ranges in older children and adolescents in sub-Saharan Africa. *Int J Cardiol.* 2017;248:409-413.
